# Supplementary material for: A Comparative Analysis of the Stomach, Gut, and Lung Microbiomes in Rattus norvegicus
Source: Microorganisms. 2023 Sep 21;11(9):2359. doi: 10.3390/microorganisms11092359 (PMC10534326; doi:10.3390/microorganisms11092359)
Supplement: Supplementary file 1 [file microorganisms-11-02359-s001.zip › Suppl. Figures legends.pdf]

Supplementary Figure legends

Supplementary Figure S1. Alpha rarefaction curves. Chao1 and Simpson indexes estimated microbial richness for all samples. The average richness of Chao1 and Simpson diversity indexes from all sequences (x-axis) are shown with color lines and lower limits of the 95% confidence interval. The number on top right side represent: gut samples (A02, A04, D02, D04, E02, E04, H04); lung (Z01, Z02, Z05, Z06, Z07, Z08, L01); stomach (A01, A03, D01, D03, E01, E03, H01).

Supplementary Figure S2. The relative microbial abundance among top 20 OTUs in each stomach, gut, and lung sample.

Supplementary Figure S3. Multivariate statistical analysis, LEfSe (p <0.05; Linear Discriminant Analysis-LDA score >3.00), analyzed differentially abundant taxa as taxonomic biomarkers of the gut (red), lung (green), and stomach (blue) samples. The relative abundance of 156 bacterial taxa was significantly higher in the lungs, followed by 50 taxa in the stomach and 27 in the gut group.

Supplementary Figure S4. Random forest analysis determined top 15 OTU biomarkers with the highest discriminatory power between gut samples (A02, A04, D02, D04, E02, E04, H04), lungs (Z01, Z02, Z05, Z06, Z07, Z08, L01), and stomach (A01, A03, D01, D03, E01, E03, H01).
