# Supplementary material for: A Comparative Analysis of the Stomach, Gut, and Lung Microbiomes in Rattus norvegicus
Source: Microorganisms. 2023 Sep 21;11(9):2359. doi: 10.3390/microorganisms11092359 (PMC10534326; doi:10.3390/microorganisms11092359)
Supplement: Supplementary file 1 [file microorganisms-11-02359-s001.zip › Suppl. Tables S1, S2, S3, S4.S5, S6, S7, S8, S9,S10.pdf]

Table S1. Number of raw reads and cleaned tags obtained from *R. norvegicus* gut, stomach, and lungs.

Sample ID	Specimens	Total raw reads	Total clean tags	Q20(%)	Q30(%)
A02	Gut	124,767	112,689	98	93.6
A04	Gut	130,049	115,352	97.8	93
D02	Gut	126,997	117,669	98	93.5
D04	Gut	124,202	114,333	98	93.5
E02	Gut	124,283	105,546	97.9	93.4
E04	Gut	125,365	107,436	97.4	92.3
H04	Gut	130,509	119,215	98	93.4
A01	Stomach	128,371	113,866	98	93.7
A03	Stomach	121,698	109,333	98	93.4
D01	Stomach	120,102	107,655	97.9	93.1
E01	Stomach	127,364	114,193	97.8	93.1
D03	Stomach	122,405	110,556	98	93.3
E03	Stomach	131,065	117,557	97.9	93.1
H01	Stomach	121,451	110,476	98	93.4
L01	Lung	125,838	103,357	98.1	93.7
Z01	Lung	125,987	112,549	97.8	93.1
Z02	Lung	123,550	114,417	98.2	94
Z05	Lung	123,725	109,861	98.1	93.8
Z06	Lung	120,422	106,576	98.4	94.5
Z07	Lung	122,492	108,152	97.9	93.3
Z08	Lung	125,168	108,701	98.2	94
**Total**	**--**	**2,625,810**	**2,339,489**	**97.97 (avg.)**	**93.44 (avg.)**

Table S2. Total number of OTUs obtained from R. *norvegicus* gut, stomach, and lungs.

Sample ID	Specimens	No. of OTUs	No. of seqs
A02	Gut	187	102,606
A04	Gut	263	96,255
D02	Gut	122	106,377
D04	Gut	168	102,454
E02	Gut	291	75,092
E04	Gut	142	98,629
H04	Gut	141	113,417
A01	Stomach	405	89,850
A03	Stomach	493	102,038
D01	Stomach	272	101,002
D03	Stomach	281	103,772
E01	Stomach	523	89,906
E03	Stomach	559	111,214
H01	Stomach	514	100,966
Z01	Lung	440	104,856
Z02	Lung	318	109,065
Z05	Lung	422	84,659
Z06	Lung	488	64,323
Z07	Lung	541	92,482
Z08	Lung	415	75,396
L01	Lung	109	70,806
**Total**	**--**	**7,094**	**1,995,165**

Supplementary Table S3. Top 15 OUTs abundance among the *R. norvegicus* gut, lungs, and stomach groups

OTUs	Gut	Lung	Stomach	Grade
OTU_1:k:Bacteria,p:Firmicutes,c:Bacilli,o:Lactobacillales,f:Lactobacillaceae,g:Lactobacillus,s:Lactobacillus johnsonii	0.380706	0.062072	0.301744	15
OTU_4:k:Bacteria,p:Firmicutes,c:Bacilli,o:Lactobacillales,f:Lactobacillaceae,g:Lactobacillus,s:Limosilactobacillus reuteri	0.140509	0.014289	0.050605	14
OTU_3:k:Bacteria,p:Proteobacteria,c:Gammaproteobacteria,o:Burkholderiales,f:Alcaligenaceae,g:Bordetella,s:Bordetella hinzii	0.031462	0.156057	0.000537	13
OTU_6:k:Bacteria,p:Firmicutes,c:Bacilli,o:Erysipelotrichales,f:Erysipelotrichaceae,g:Turicibacter,s:Turicibacter sp. LA61	0.142502	0.013454	0.009152	12
OTU_5:k:Bacteria,p:Firmicutes,c:Bacilli,o:Lactobacillales,f:Lactobacillaceae,g:Lactobacillus,s:Lactobacillus intestinalis	0.011561	0.011139	0.101311	11

OTU_10:k:Bacteria,p:Firmicutes,c:Bacilli,o:Lactobacillales,f:Lactobacillaceae,g:Lactobacillus,s:Lactobacillus murinus	0.01064	0.057697	0.026284	10
OTU_7:k:Bacteria,p:Actinobacteriota,c:Actinobacteria,o:Bifidobacteriales,f:Bifidobacteriaceae,g:Bifidobacterium,s:Bifidobacterium faecale	0.002024	0.050212	0.027289	9
OTU_13:k:Bacteria,p:Firmicutes,c:Bacilli,o:Lactobacillales,f:Streptococcaceae,g:Streptococcus,s:Streptococcus gallolyticus	0.003058	0.040874	0.03094	8
OTU_1149:k:Bacteria,p:Firmicutes,c:Bacilli,o:Lactobacillales,f:Lactobacillaceae,g:Lactobacillus,s:Lactobacillus acidophilus	0.035032	0.012738	0.022874	7
OTU_22:k:Bacteria,p:Bacteroidota,c:Bacteroidia,o:Bacteroidales,f:Prevotellaceae,g:Prevotella,s:uncultured Prevotella	9.07E-05	0.044501	0.008811	6
OTU_9:k:Bacteria,p:Campilobacterota,c:Campylobacteria,o:Campylobacterales,f:Helicobacteraceae,g:Helicobacter,s:Helicobacter apodemus	0.02684	0.003566	0.021162	5
OTU_8:k:Bacteria,p:Campilobacterota,c:Campylobacteria,o:Campylobacterales,f:Helicobacteraceae,g:Helicobacter,s:Helicobacter muridarum	0.020055	0.001105	0.028215	4
OTU_15:k:Bacteria,p:Firmicutes,c:Bacilli,o:Staphylococcales,f:Staphylococcaceae,g:Macrococcus,s:Macrococcus goetzii	0.017565	0.023584	0.003415	3
OTU_12:k:Bacteria,p:Proteobacteria,c:Gammaproteobacteria,o:Enterobacterales,f:Enterobacteriaceae,g:Escherichia-Shigella,s:E. coli	0.016709	0.011207	0.015924	2
OTU_11:k:Bacteria,p:Proteobacteria,c:Gammaproteobacteria,o:Burkholderiales,f:Burkholderiaceae,g:Ralstonia,s:Ralstonia insidiosa	0.043501	6.65E-06	2.86E-05	1

Keynote: k: kingdom, p: phylum, c: class, o: order, f: family, g: genus, s: species

Supplementary Table S4. Details of taxonomic levels among the top 20 OTUs in the *Rattus norvegicus* gut, lung, and stomach.

**OTUs**	**Gut**	**Lung**	**Stomach**
OTU_1:k:Bacteria,p:Firmicutes,c:Bacilli,o:Lactobacillales,f:Lactobacillaceae,g:Lactobacillus,s:Lactobacillus johnsonii	0.380706	0.062072	0.301744
OTU_4:k:Bacteria,p:Firmicutes,c:Bacilli,o:Lactobacillales,f:Lactobacillaceae,g:Lactobacillus,s:Limosilactobacillus reuteri	0.140509	0.014289	0.050605
OTU_3:k:Bacteria,p:Proteobacteria,c:Gammaproteobacteria,o:Burkholderiales,f:Alcaligenaceae,g:Bordetella, s:Bordetella hinzii	0.031462	0.156057	0.000537
OTU_6:k:Bacteria,p:Firmicutes,c:Bacilli,o:Erysipelotrichales,f:Erysipelotrichaceae,g:Turicibacter,s:Turicibacter sp. LA61	0.142502	0.013454	0.009152
OTU_5:k:Bacteria,p:Firmicutes,c:Bacilli,o:Lactobacillales,f:Lactobacillaceae,g:Lactobacillus,s:Lactobacillus intestinalis	0.011561	0.011139	0.101311
OTU_10:k:Bacteria,p:Firmicutes,c:Bacilli,o:Lactobacillales,f:Lactobacillaceae,g:Lactobacillus,s:Lactobacillus murinus	0.01064	0.057697	0.026284
OTU_7:k:Bacteria,p:Actinobacteriota,c:Actinobacteria,o:Bifidobacteriales,f:Bifidobacteriaceae,g:Bifidobacterium, s:Bifidobacterium faecale	0.002024	0.050212	0.027289
OTU_13:k:Bacteria,p:Firmicutes,c:Bacilli,o:Lactobacillales,f:Streptococcaceae,g:Streptococcus,s:Streptococcus gallolyticus	0.003058	0.040874	0.03094
OTU_1149:k:Bacteria,p:Firmicutes,c:Bacilli,o:Lactobacillales,f:Lactobacillaceae,g:Lactobacillus,s:Lactobacillus acidophilus	0.035032	0.012738	0.022874
OTU_9:k:Bacteria,p:Campilobacterota,c:Campylobacteria,o:Campylobacterales,f:Helicobacteraceae,g:Helicobacter,s:Helicobacter apodemus	0.02684	0.003566	0.021162
OTU_8:k:Bacteria,p:Campilobacterota,c:Campylobacteria,o:Campylobacterales,f:Helicobacteraceae,g:Helicobacter,s:Helicobacter muridarum	0.020055	0.001105	0.028215
OTU_22:k:Bacteria,p:Bacteroidota,c:Bacteroidia,o:Bacteroidales,f:Prevotellaceae,g:Prevotella, s:uncultured Prevotella	9.07E-05	0.044501	0.008811

OTU_11:k:Bacteria,p:Proteobacteria,c:Gammaproteobacteria,o:Burkholderiales,f:Burkholderiaceae,g:Ralstonia, s:Ralstonia insidiosa	0.043501	6.65E-06	2.86E-05
OTU_12:k:Bacteria,p:Proteobacteria,c:Gammaproteobacteria,o:Enterobacterales,f:Enterobacteriaceae,g:Escherichia-Shigella, s:E. coli	0.016709	0.011207	0.015924
OTU_15:k:Bacteria,p:Firmicutes,c:Bacilli,o:Staphylococcales,f:Staphylococcaceae,g:Macrococcus,s:Macrococcus goetzii	0.017565	0.023584	0.003415
OTU_14:k:Bacteria,p:Bacteroidota,c:Bacteroidia,o:Bacteroidales,f:Porphyromonadaceae,g:Porphyromonas,s:uncultured bacterium	3.74E-05	0.034643	0.000664
OTU_16:k:Bacteria,p:Actinobacteriota,c:Coriobacteriia,o:Coriobacteriales,f:Atopobiaceae,g:Olsenella,s:uncultured bacterium	0.000933	0.01331	0.018105
OTU_23:k:Bacteria,p:Proteobacteria,c:Gammaproteobacteria,o:Pasteurellales,f:Pasteurellaceae,g:Rodentibacter,s:Pasteurellaceae bacterium	0.000351	0.028348	0.005274
OTU_17:k:Bacteria,p:Firmicutes,c:Bacilli,o:Lactobacillales,f:Lactobacillaceae,g:Lactobacillus, s:Limosilactobacillus mucosae	0.003985	0.010735	0.016678
OTU_18:k:Bacteria,p:Actinobacteriota,c:Actinobacteria,o:Bifidobacteriales,f:Bifidobacteriaceae,g:Bifidobacterium,s:Bifidobacterium pseudolongum	0.001172	0.017402	0.009252

Keynote: k: kingdom, p: phylum, c: class, o: order, f: family, g: genus, s: species

Supplementary Table S5. Values of heatmap based on Bray-Curtis distance.

Samples	L01	A01	A03	D01	E01	D03	E03	H01	Z01	Z02	Z05	Z06	Z07	Z08	A02	A04	D02	D04	E02	E04	H04
**LO1**	0	0.958	0.980	0.986	0.992	0.926	0.921	0.958	0.980	0.988	0.964	0.972	0.976	0.968	0.989	0.983	0.994	0.927	0.981	0.996	0.989
**A01**	0.958	0	0.534	0.617	0.600	0.504	0.535	0.560	0.412	0.941	0.571	0.573	0.584	0.506	0.484	0.611	0.736	0.689	0.714	0.864	0.548
**A03**	0.980	0.534	0	0.422	0.676	0.483	0.434	0.561	0.741	0.931	0.699	0.651	0.704	0.674	0.418	0.477	0.650	0.656	0.776	0.919	0.384
**D01**	0.986	0.617	0.422	0	0.706	0.542	0.453	0.461	0.835	0.944	0.756	0.670	0.784	0.750	0.524	0.328	0.426	0.536	0.751	0.915	0.469
**E01**	0.992	0.600	0.676	0.706	0	0.664	0.669	0.553	0.626	0.961	0.518	0.515	0.542	0.589	0.767	0.758	0.768	0.789	0.673	0.954	0.749
**D03**	0.926	0.504	0.483	0.542	0.664	0	0.396	0.517	0.678	0.932	0.601	0.541	0.610	0.560	0.533	0.524	0.683	0.667	0.726	0.927	0.535
**E03**	0.921	0.535	0.434	0.453	0.669	0.396	0	0.511	0.745	0.922	0.702	0.591	0.651	0.651	0.437	0.367	0.587	0.601	0.723	0.902	0.443
**H01**	0.958	0.560	0.561	0.461	0.553	0.517	0.511	0	0.680	0.942	0.620	0.567	0.548	0.612	0.663	0.637	0.669	0.704	0.686	0.928	0.666
**Z01**	0.980	0.412	0.741	0.835	0.626	0.678	0.745	0.680	0	0.946	0.537	0.586	0.497	0.576	0.740	0.854	0.853	0.862	0.740	0.849	0.816
**Z02**	0.988	0.941	0.931	0.944	0.961	0.932	0.922	0.942	0.946	0	0.958	0.955	0.949	0.962	0.958	0.931	0.954	0.708	0.931	0.960	0.967
**Z05**	0.964	0.571	0.699	0.756	0.518	0.601	0.702	0.620	0.537	0.958	0	0.348	0.363	0.440	0.823	0.821	0.805	0.832	0.594	0.950	0.826
**Z06**	0.972	0.573	0.651	0.670	0.515	0.541	0.591	0.567	0.586	0.955	0.348	0	0.384	0.365	0.775	0.716	0.721	0.745	0.572	0.948	0.773
**Z07**	0.976	0.584	0.704	0.784	0.542	0.610	0.651	0.548	0.497	0.949	0.363	0.384	0	0.462	0.832	0.828	0.813	0.845	0.674	0.943	0.835
**Z08**	0.968	0.506	0.674	0.750	0.589	0.560	0.651	0.612	0.576	0.962	0.440	0.365	0.462	0	0.730	0.770	0.816	0.788	0.690	0.954	0.733
**A02**	0.989	0.484	0.418	0.524	0.767	0.533	0.437	0.663	0.740	0.958	0.823	0.775	0.832	0.730	0	0.376	0.653	0.647	0.806	0.773	0.218

**A04**	0.983	0.611	0.477	0.328	0.758	0.524	0.367	0.637	0.854	0.931	0.821	0.716	0.828	0.770	0.376	0	0.406	0.432	0.728	0.889	0.365
**D02**	0.994	0.736	0.650	0.426	0.768	0.683	0.587	0.669	0.853	0.954	0.805	0.721	0.813	0.816	0.653	0.406	0	0.578	0.744	0.895	0.646
**D04**	0.927	0.689	0.656	0.536	0.789	0.667	0.601	0.704	0.862	0.708	0.832	0.745	0.845	0.788	0.647	0.432	0.578	0	0.634	0.923	0.645
**E02**	0.981	0.714	0.776	0.751	0.673	0.726	0.723	0.686	0.740	0.931	0.594	0.572	0.674	0.690	0.806	0.728	0.744	0.634	0	0.923	0.783
**E04**	0.996	0.864	0.919	0.915	0.954	0.927	0.902	0.928	0.849	0.960	0.950	0.948	0.943	0.954	0.773	0.889	0.895	0.923	0.923	0	0.907
**H04**	0.989	0.548	0.384	0.469	0.749	0.535	0.443	0.666	0.816	0.967	0.826	0.773	0.835	0.733	0.218	0.365	0.646	0.645	0.783	0.907	0

A01 (stomach); A03 (stomach); D01 (stomach); E01 (stomach); D03 (stomach); E03 (stomach); H01 (stomach)
L01 (lung); Z01 (lung); Z02 (lung); Z05 (lung); Z06 (lung); Z07 (lung); Z08 (lung)
A02 (gut); A04 (gut); D02 (gut); D04 (gut); E02 (gut); E04 (gut); H04 (gut)

Supplementary Table S6. Values of heatmap based on Euclidean distance.

Samples	L01	A01	A03	D01	E01	D03	E03	H01	Z01	Z02	Z05	Z06	Z07	Z08	A02	A04	D02	D04	E02	E04	H04
**LO1**	0	1.404	1.412	1.413	1.412	1.394	1.388	1.402	1.409	1.412	1.4015	1.408	1.409	1.405	1.412	1.411	1.413	1.400	1.411	1.414	1.413
**A01**	1.404	0	0.727	0.833	0.806	0.577	0.613	0.919	0.852	1.399	0.9246	0.841	1.006	0.596	0.565	0.677	1.038	0.905	1.1767	1.244	0.626
**A03**	1.412	0.727	0	0.637	0.931	0.623	0.533	0.835	1.159	1.401	1.1093	0.925	1.161	0.823	0.470	0.598	0.988	0.876	1.205	1.358	0.477
**D01**	1.413	0.833	0.637	0	0.889	0.653	0.531	0.502	1.217	1.402	1.0681	0.861	1.146	0.900	0.697	0.507	0.712	0.822	1.168	1.360	0.666
**E01**	1.412	0.806	0.931	0.889	0	0.830	0.836	0.846	1.044	1.398	0.788	0.802	0.812	0.780	0.900	0.901	1.089	1.045	1.135	1.372	0.884
**D03**	1.394	0.577	0.623	0.653	0.830	0	0.387	0.807	1.083	1.398	0.972	0.775	1.029	0.646	0.483	0.511	0.992	0.825	1.166	1.347	0.488
**E03**	1.388	0.613	0.533	0.531	0.836	0.387	0	0.792	1.130	1.400	1.062	0.812	1.089	0.731	0.332	0.286	0.883	0.741	1.167	1.335	0.326
**H01**	1.402	0.919	0.835	0.502	0.846	0.807	0.792	0	1.190	1.403	1.029	0.944	1.077	0.947	0.906	0.874	1.064	1.042	1.213	1.368	0.902
**Z01**	1.409	0.852	1.159	1.217	1.044	1.083	1.130	1.190	0	1.399	0.980	1.023	0.907	0.912	1.125	1.183	1.279	1.236	1.295	1.200	1.170
**Z02**	1.412	1.399	1.401	1.402	1.398	1.398	1.400	1.403	1.399	0	1.397	1.397	1.396	1.396	1.401	1.385	1.401	1.002	1.367	1.407	1.401
**Z05**	1.401	0.924	1.109	1.068	0.788	0.972	1.062	1.029	0.980	1.397	0	0.554	0.559	0.716	1.129	1.108	1.149	1.170	1.060	1.372	1.127
**Z06**	1.408	0.841	0.925	0.861	0.802	0.775	0.812	0.944	1.023	1.397	0.554	0	0.725	0.604	0.919	0.848	0.954	0.995	0.954	1.356	0.918
**Z07**	1.409	1.006	1.161	1.146	0.812	1.029	1.089	1.077	0.907	1.396	0.559	0.725	0	0.824	1.171	1.161	1.211	1.210	1.196	1.378	1.171
**Z08**	1.405	0.596	0.823	0.900	0.780	0.646	0.731	0.947	0.912	1.396	0.716	0.604	0.824	0	0.767	0.811	1.089	0.984	1.119	1.355	0.767
**A02**	1.412	0.565	0.470	0.697	0.900	0.483	0.332	0.906	1.125	1.401	1.129	0.919	1.171	0.767	0	0.444	1.003	0.808	1.188	1.206	0.303
**A04**	1.411	0.677	0.598	0.507	0.901	0.511	0.286	0.874	1.183	1.385	1.108	0.848	1.161	0.811	0.444	0	0.678	0.654	1.139	1.348	0.439
**D02**	1.413	1.038	0.988	0.712	1.089	0.992	0.883	1.064	1.279	1.401	1.149	0.954	1.211	1.089	1.003	0.678	0	0.864	1.157	1.366	1.00

**D04**	1.400	0.905	0.876	0.822	1.045	0.825	0.741	1.042	1.236	1.002	1.170	0.995	1.210	0.984	0.808	0.654	0.864	0	0.987	1.357	0.808
**E02**	1.411	1.176	1.205	1.168	1.135	1.166	1.167	1.213	1.295	1.367	1.060	0.954	1.196	1.119	1.188	1.139	1.157	0.987	0	1.375	1.178
**E04**	1.414	1.244	1.358	1.360	1.372	1.347	1.335	1.368	1.200	1.407	1.372	1.356	1.378	1.355	1.206	1.348	1.366	1.357	1.375	0	1.352
**H04**	1.413	0.626	0.477	0.666	0.884	0.488	0.326	0.902	1.170	1.401	1.127	0.918	1.171	0.7675	0.303	0.439	1.002	0.808	1.178	1.352	0

A01 (stomach); A03 (stomach); D01 (stomach); E01 (stomach); D03 (stomach); E03 (stomach); H01 (stomach)
L01 (lung); Z01 (lung); Z02 (lung); Z05 (lung); Z06 (lung); Z07 (lung); Z08 (lung)
A02 (gut); A04 (gut); D02 (gut); D04 (gut); E02 (gut); E04 (gut); H04 (gut)

Supplementary Table S7. Unique 202 OTUs in lung samples, contributing to variance in the overall sequences obtained.

No.	OUT descriptions
1	OTU_583:k:Bacteria,p:Firmicutes,c:Clostridia,o:Peptostreptococcales-Tissierellales,g:Parvimonas,s:Uncultured bacterium
2	OTU_213:k:Bacteria,p:Proteobacteria,c:Gammaproteobacteria,o:Pseudomonadales,f:Moraxellaceae,g:Acinetobacter,s:Acinetobacter towneri
3	OTU_1306:k:Bacteria,p:Bacteroidota,c:Bacteroidia,o:Chitinophagales,f:Chitinophagaceae,g:Cnuella,s:Uncultured bacterium
4	OTU_430:k:Bacteria,p:Fusobacteriota,c:Fusobacteriia,o:Fusobacteriales,f:Fusobacteriaceae,g:Cetobacterium,s:Cetobacterium somerae
5	OTU_814:k:Bacteria,p:Proteobacteria,c:Gammaproteobacteria,o:Pasteurellales,f:Pasteurellaceae,s:[Actinobacillus] muris
6	OTU_618:k:Bacteria,p:Bacteroidota,c:Bacteroidia,o:Bacteroidales,f:Porphyromonadaceae,g:Porphyromonas
7	OTU_1254:k:Bacteria,p:Bacteroidota,c:Bacteroidia,o:Bacteroidales,f:Prevotellaceae,g:Prevotella
8	OTU_3288:k:Bacteria,p:Firmicutes,c:Bacilli,o:Mycoplasmatales,f:Mycoplasmataceae,g:Mycoplasma,s:Uncultured Mycoplasma
9	OTU_3537:k:Bacteria,p:Proteobacteria,c:Gammaproteobacteria
10	OTU_930:k:Bacteria,p:Firmicutes,c:Clostridia,o:Oscillospirales,f:[Eubacterium]-coprostanoligenes group
11	OTU_1423:k:Bacteria,p:Bacteroidota,c:Bacteroidia,o:Bacteroidales,f:Prevotellaceae,g:Prevotella
12	OTU_1459:k:Bacteria,p:Proteobacteria,c:Gammaproteobacteria,o:Pseudomonadales,f:Moraxellaceae,g:Moraxella,s:uncultured bacterium
13	OTU_4466:k:Bacteria,p:Firmicutes,c:Clostridia,o:Peptostreptococcales-Tissierellales,g:Gallicola,s:Miniphocibacter massiliensis
14	OTU_3367:k:Bacteria,p:Proteobacteria,c:Gammaproteobacteria,o:Pasteurellales,f:Pasteurellaceae,s:Rodentibacter pneumotropicus
15	OTU_3278:k:Bacteria,p:Proteobacteria,c:Gammaproteobacteria,o:Enterobacterales,f:Enterobacteriaceae,g:Plesiomonas
16	OTU_4390:k:Bacteria,p:Proteobacteria,c:Gammaproteobacteria,o:Pasteurellales,f:Pasteurellaceae,s:Pasteurellaceae bacterium
17	OTU_1688:k:Bacteria,p:Chloroflexi,c:Chloroflexia,o:Thermomicrobiales,f:JG30-KF-CM45

18 OTU_3070:k:Bacteria,p:Bacteroidota,c:Bacteroidia,o:Flavobacteriales,f:Weeksellaceae,g:Bergeyella
19 OTU_2779:k:Bacteria,p:Actinobacteriota,c:Actinobacteria,o:Propionibacteriales,f:Propionibacteriaceae,g:Micropruina
20 OTU_4124:k:Bacteria,p:Firmicutes,c:Clostridia,o:Christensenellales,f:Christensenellaceae,g:Christensenellaceae R-7 group,s:uncultured bacterium
21 OTU_4257:k:Bacteria,p:Proteobacteria,c:Gammaproteobacteria,o:Burkholderiales,f:Alcaligenaceae,g:uncultured,s:uncultured bacterium
22 OTU_1405:k:Bacteria,p:Firmicutes,c:Clostridia,o:Oscillospirales,f:Oscillospiraceae,g:UCG-003,s:uncultured bacterium
23 OTU_2107:k:Bacteria,p:Cyanobacteria,c:Cyanobacteriia,o:Cyanobacteriales,f:Chroococcidiopsaceae,g:Chroococcidiopsis SAG-2023,s:Chroococcidiopsis BB79.2
24 OTU_3419:k:Bacteria,p:Actinobacteriota,c:Thermoleophilia,o:Gaiellales,f:Gaiellaceae,g:Gaiella,s:uncultured bacterium
25 OTU_3309:k:Bacteria,p:Firmicutes,c:Clostridia,o:Monoglobales,f:Monoglobaceae,g:Monoglobus,s:Uncultured bacterium
26 OTU_2138:k:Bacteria,p:Proteobacteria,c:Alphaproteobacteria,o:Rhizobiales,f:Rhizobiaceae
27 OTU_2171:k:Bacteria,p:Proteobacteria,c:Gammaproteobacteria,o:Aeromonadales,f:Succinivibrionaceae,g:Succinivibrio,s:Uncultured Succinivibrio
28 OTU_4104:k:Bacteria,p:Firmicutes,c:Clostridia,o:Clostridiales,f:Clostridiaceae,g:Clostridium sensu stricto 1,s:uncultured bacterium
29 OTU_4321:k:Bacteria,p:Patescibacteria,c:Saccharimonadia,o:Saccharimonadales, s:Uncultured bacterium
30 OTU_2114:k:Bacteria,p:Deinococcota,c:Deinococci,o:Deinococcales,f:Deinococcaceae,g:Deinococcus
31 OTU_1726:k:Bacteria,p:Proteobacteria,c:Gammaproteobacteria,o:Pseudomonadales,f:Moraxellaceae,g:Psychrobacter
32 OTU_2600:k:Bacteria,p:Proteobacteria,c:Gammaproteobacteria,o:Burkholderiales,f:Oxalobacteraceae,g:Massilia,s:Uncultured
33 OTU_2629:k:Bacteria,p:Bacteroidota,c:Bacteroidia,o:Sphingobacteriales,f:Sphingobacteriaceae,g:Solitalea
34 OTU_2253:k:Bacteria,p:Proteobacteria,c:Gammaproteobacteria,o:Burkholderiales,f:Oxalobacteraceae,g:Undibacterium,s:Undibacterium macrobrachii
35 OTU_4488:k:Bacteria,p:Firmicutes,c:Clostridia,o:Lachnospirales,f:Lachnospiraceae,g:Lachnospira,s:uncultured bacterium
36 OTU_3979:k:Bacteria,s:Uncultured bacterium
37 OTU_4114:k:Bacteria,p:Firmicutes,c:Clostridia,o:Oscillospirales,f:Oscillospiraceae,g:NK4A214 group,s:uncultured bacterium
38 OTU_3773:k:Bacteria,p:Proteobacteria,c:Alphaproteobacteria,o:Rhizobiales,f:Xanthobacteraceae
39 OTU_893:k:Bacteria,p:Proteobacteria,c:Alphaproteobacteria,o:Rhodobacterales,f:Rhodobacteraceae
40 OTU_4434:k:Bacteria,p:Bacteroidota,c:Bacteroidia,o:Bacteroidales,f:Prevotellaceae,g:Prevotella,s:uncultured bacterium
41 OTU_3308:k:Bacteria,p:Proteobacteria,c:Alphaproteobacteria,o:Azospirillales,f:Azospirillaceae,g:Skermanella
42 OTU_3273:k:Bacteria,p:Firmicutes,c:Clostridia,o:Oscillospirales,f:Ruminococcaceae,g:Ruminococcus,s:uncultured bacterium
43 OTU_2885:k:Bacteria,p:Firmicutes,c:Clostridia,o:Clostridiales,f:Clostridiaceae,g:Candidatus Arthromitus
44 OTU_1075:k:Bacteria,p:Proteobacteria,c:Alphaproteobacteria,o:Rhizobiales,f:Rhizobiaceae
45 OTU_1343:k:Bacteria,p:Firmicutes,c:Clostridia,o:Oscillospirales,f:Ruminococcaceae,g:Phocea,s:Phocea massiliensis
46 OTU_2686:k:Bacteria,p:Proteobacteria,c:Alphaproteobacteria,o:Rhodobacterales,f:Rhodobacteraceae,g:uncultured,s:uncultured bacterium
47 OTU_4110:k:Bacteria,p:Firmicutes,c:Clostridia,o:Oscillospirales,f:UCG-010
48 OTU_4065:k:Bacteria,p:Bacteroidota,c:Bacteroidia,o:Bacteroidales,f:Bacteroidaceae,g:Bacteroides,s:uncultured bacterium

49 OTU_515:k:Bacteria,p:Proteobacteria,c:Gammaproteobacteria,o:Enterobacterales,f:Morganellaceae,g:Proteus

50 OTU_3783:k:Bacteria,p:Bacteroidota,c:Bacteroidia,o:Bacteroidales,f:Prevotellaceae,g:Prevotellaceae UCG-004,s:uncultured bacterium

51 OTU_2087:k:Bacteria,p:Firmicutes,c:Clostridia,o:Lachnospirales,f:Lachnospiraceae,g:Lachnospira,s:Kineothrix

52 OTU_2690:k:Bacteria,p:Bacteroidota,c:Bacteroidia,o:Bacteroidales,f:Bacteroidales RF16 group

53 OTU_3171:k:Archaea,p:Halobacterota,c:Halobacteria,o:Halobacterales,f:Haloferacaceae,g:Haloquadratum

54 OTU_2261:k:Bacteria,p:Proteobacteria,c:Alphaproteobacteria,o:Rhodobacterales,f:Rhodobacteraceae,g:Rubellimicrobium,s:uncultured bacterium

55 OTU_2760:k:Bacteria,p:Patescibacteria,c:Gracilibacteria,o:Absconditabacteriales SR1, s:Uncultured bacterium

56 OTU_4013:k:Bacteria

57 OTU_3266:k:Bacteria

58 OTU_3168:k:Bacteria,p:Firmicutes,c:Bacilli,o:Mycoplasmatales,f:Mycoplasmataceae,g:Ureaplasma,s:uncultured bacterium

59 OTU_1646:k:Bacteria,p:Bacteroidota,c:Bacteroidia,o:Bacteroidales,f:Muribaculaceae

60 OTU_498:k:Bacteria,p:Proteobacteria,c:Alphaproteobacteria,o:Caulobacterales,f:Caulobacteraceae,g:Caulobacter

61 OTU_1307:k:Bacteria,p:Proteobacteria,c:Alphaproteobacteria,o:Rhodobacterales,f:Rhodobacteraceae,g:HIMB11

62 OTU_1268:k:Bacteria,p:Firmicutes,c:Clostridia,o:Lachnospirales,f:Lachnospiraceae g:Lachnospira,s:uncultured bacterium

63 OTU_3144:k:Bacteria,p:Patescibacteria,c:Dojkabacteria

64 OTU_1096:k:Bacteria,p:Actinobacteriota,c:Actinobacteria,o:Propionibacteriales,f:Propionibacteriaceae,g:Cutibacterium

65 OTU_1639:k:Bacteria,p:Patescibacteria,c:Saccharimonadia,o:Saccharimonadales

66 OTU_2024:k:Bacteria,p:Proteobacteria,c:Alphaproteobacteria,o:Sphingomonadales,f:Sphingomonadaceae

67 OTU_1348:k:Bacteria,p:Firmicutes,c:Clostridia

68 OTU_3887:k:Bacteria,p:Firmicutes,c:Clostridia,o:Lachnospirales,f:Lachnospiraceae,g:Lachnospira,s:Lachnospiraceae bacterium S37-30-1

69 OTU_2811:k:Bacteria,p:Firmicutes,c:Clostridia,o:Peptostreptococcales-Tissierellales

70 OTU_4503:k:Bacteria,p:Proteobacteria,c:Gammaproteobacteria,o:Pseudomonadales,f:Moraxellaceae,g:Acinetobacter

71 OTU_1251:k:Bacteria,p:Firmicutes,c:Clostridia,o:Clostridia vadinBB60 group

72 OTU_1566:k:Bacteria,p:Bacteroidota,c:Bacteroidia,o:Bacteroidales,f:Muribaculaceae

73 OTU_2903:k:Bacteria,p:Acidobacteriota,c:Thermoanaerobaculia,o:Thermoanaerobaculales,f:Thermoanaerobaculaceae,g:Subgroup-1

74 OTU_2356:k:Bacteria,p:Firmicutes,c:Clostridia

75 OTU_1535:k:Bacteria,p:Acidobacteriota,c:Vicinamibacteria,o:Subgroup-17

76 OTU_2325:k:Bacteria,p:Proteobacteria,c:Alphaproteobacteria,o:Rhodobacterales,f:Rhodobacteraceae,g:Rhodobacter

77 OTU_1386:k:Bacteria,p:Firmicutes,c:Clostridia,o:Clostridia UCG-014

78 OTU_1493:k:Bacteria,p:Firmicutes,c:Bacilli,o:Brevibacillales,f:Brevibacillaceae,g:Brevibacillus,s:bacterium YC-LK-LKJ16

79 OTU_2349:k:Bacteria,p:Bacteroidota,c:Bacteroidia,o:Bacteroidales,f:Prolixibacteraceae,g:WCHB1-32

80 OTU_993:k:Bacteria,p:Actinobacteriota,c:Acidimicrobiia,o:IMCC26256

81 OTU_1804:k:Bacteria,p:Firmicutes,c:Clostridia,o:Christensenellales,f:Christensenellaceae,g:Christensenellaceae-R-7-group

82 OTU_3220:k:Bacteria,p:Proteobacteria,c:Gammaproteobacteria,o:Enterobacterales,f:Yersiniaceae,g:Rahnella,s:Rahnella aquatilis

83 OTU_3468:k:Bacteria,p:Proteobacteria,c:Gammaproteobacteria,o:Enterobacterales

84 OTU_1232:k:Bacteria,p:Bacteroidota,c:Bacteroidia,o:Bacteroidales,f:Barnesiellaceae,g:Barnesiella

85 OTU_946:k:Bacteria,p:Bacteroidota,c:Bacteroidia,o:Flavobacteriales,f:Weeksellaceae,g:Empedobacter,s:Empedobacter brevis

86 OTU_4096:k:Bacteria,p:Firmicutes,c:Clostridia,o:Clostridiales,f:Caloramatoraceae

87 OTU_1908:k:Bacteria,p:Proteobacteria,c:Gammaproteobacteria,o:Burkholderiales,f:Rhodocyclaceae,g:uncultured,s:uncultured bacterium

88 OTU_1909:k:Bacteria,p:Firmicutes,c:Clostridia

89 OTU_1312:k:Bacteria,p:Firmicutes,c:Negativicutes,o:Veillonellales-Selenomonadales,f:Selenomonadaceae,g:Selenomonas

90 OTU_2859:k:Bacteria,p:Proteobacteria,c:Alphaproteobacteria,o:Rhizobiales,f:Hyphomicrobiaceae,g:Hyphomicrobium

91 OTU_940:k:Bacteria,p:Firmicutes,c:Clostridia,o:Oscillospirales,f:Ruminococcaceae,g:Paludicola,s:Paludicola psychrotolerans

92 OTU_1100:k:Bacteria,p:Bacteroidota,c:Bacteroidia,o:Bacteroidales,f:Porphyromonadaceae,g:Porphyromonas,s:Porphyromonas pogonae

93 OTU_2060:k:Bacteria,p:Firmicutes,c:Limnochordia

94 OTU_2899:k:Bacteria,p:Acidobacteriota,c:Blastocatellia,o:11-24

95 OTU_2835:k:Bacteria,p:Proteobacteria,c:Gammaproteobacteria,o:Xanthomonadales,f:Xanthomonadaceae,g:Pseudoxanthomonas

96 OTU_1992:k:Bacteria,p:Sumerlaeota,c:Sumerlaeia,o:Sumerlaeales,f:Sumerlaeaceae,g:Sumerlaea,s:uncultured bacterium

97 OTU_3594:k:Bacteria,p:Actinobacteriota,c:Actinobacteria,o:Micromonosporales,f:Micromonosporaceae,g:Micromonospora

98 OTU_3741:k:Bacteria,p:Firmicutes,c:Negativicutes,o:Veillonellales-Selenomonadales,f:Selenomonadaceae,g:Selenomonas

99 OTU_2791:k:Bacteria,p:Bacteroidota,c:Ignavibacteria,o:Ignavibacteriales

100 OTU_3552:k:Bacteria,p:Actinobacteriota,c:Actinobacteria,o:Propionibacteriales,f:Propionibacteriaceae,g:Luteococcus,s:Luteococcus japonicus

101 OTU_2770:k:Bacteria,p:Bacteroidota,c:Bacteroidia,o:Sphingobacteriales,f:Sphingobacteriaceae,g:Sphingobacterium,s:uncultured bacterium

102 OTU_3711:k:Bacteria,p:Armatimonadota,c:Fimbriimonadia,o:Fimbriimonadales,f:Fimbriimonadaceae

103 OTU_2974:k:Bacteria,p:Proteobacteria,c:Gammaproteobacteria,o:Pseudomonadales,f:Moraxellaceae,g:Acinetobacter,s:Acinetobacter-92012

104 OTU_1248:k:Bacteria,p:Actinobacteriota,c:Actinobacteria,o:Frankiales,f:Geodermatophilaceae,g:Geodermatophilus

105 OTU_1122:k:Bacteria,p:Desulfobacterota,c:Desulfobulbia,o:Desulfobulbales,f:Desulfocapsaceae

106 OTU_1261:k:Bacteria,p:Fusobacteriota,c:Fusobacteriia,o:Fusobacteriales,f:Leptotrichiaceae,g:Leptotrichia

107 OTU_1270:k:Bacteria,p:Firmicutes,c:Clostridia,o:Oscillospirales,f:Butyricicoccaceae,g:UCG-009,s:uncultured bacterium

108 OTU_1937:k:Bacteria,p:Firmicutes,c:Clostridia,o:Oscillospirales,f:UCG-010

109 OTU_1503:k:Bacteria,p:Firmicutes,c:Clostridia,o:Oscillospirales,f:Ruminococcaceae

110 OTU_1516:k:Bacteria,p:Chloroflexi,c:Anaerolineae,o:Caldilineales,f:Caldilineaceae,g:uncultured,s:uncultured bacterium

111 OTU_2027:k:Bacteria,p:Firmicutes,c:Bacilli,o:Lactobacillales,f:Streptococcaceae,g:Streptococcus,s:Streptococcus parauberis

112 OTU_3681:k:Bacteria,p:Bacteroidota,c:Bacteroidia,o:Bacteroidales,f:Prevotellaceae,g:Prevotella,s:uncultured bacterium

113 OTU_2818:k:Bacteria,p:Bacteroidota,c:Bacteroidia,o:Bacteroidales,f:Bacteroidaceae,g:Bacteroides

114 OTU_690:k:Bacteria,p:Bacteroidota,c:Bacteroidia,o:Bacteroidales,f:Marinifilaceae,g:Butyricimonas

115 OTU_1277:k:Bacteria,p:Actinobacteriota,c:Acidimicrobiia,o:Microtrichales,f:Ilumatobacteraceae,g:CL500-29 marine,s:uncultured bacterium

116 OTU_3551:k:Bacteria,p:Firmicutes,c:Clostridia,o:Oscillospirales,f:Oscillospiraceae,g:Colidextribacter,s:uncultured bacterium

117 OTU_3556:k:Bacteria,p:Bacteroidota,c:Bacteroidia,o:Bacteroidales,f:Dysgonomonadaceae,g:Dysgonomonas

118 OTU_1370:k:Bacteria,p:Deinococcota,c:Deinococci,o:Thermales,f:Thermaceae,g:Meiothermus

119 OTU_1565:k:Bacteria,p:Planctomycetota,c:Planctomycetes,o:Gemmatales,f:Gemmataceae,g:uncultured

120 OTU_2122:k:Bacteria,p:Bacteroidota,c:Bacteroidia,o:Cytophagales,f:Cyclobacteriaceae,g:Algoriphagus,s:uncultured bacterium

121 OTU_3294:k:Bacteria,p:Proteobacteria,c:Gammaproteobacteria,o:Diplorickettsiales,f:Diplorickettsiaceae,g:Rickettsiella,s:Rickettsiella agriotidis

122 OTU_2321:k:Bacteria,p:Chloroflexi,c:Anaerolineae,o:SBR1031

123 OTU_913:k:Bacteria,p:Firmicutes,c:Bacilli,o:Lactobacillales,f:Carnobacteriaceae,g:Granulicatella

124 OTU_3897:k:Bacteria,p:Proteobacteria,c:Gammaproteobacteria,o:Burkholderiales,f:Oxalobacteraceae,g:Massilia,s:Uncultured Oxalobacteraceae

125 OTU_1483:k:Bacteria,p:Proteobacteria,c:Gammaproteobacteria,o:Pasteurellales,f:Pasteurellaceae,g:Haemophilus,s:Haemophilus haemolyticus

126 OTU_1422:k:Bacteria,p:Actinobacteriota,c:Acidimicrobiia,o:Actinomarinales,f:Actinomarinaceae,g:Candidatus Actinomarina,s:uncultured bacterium

127 OTU_1657:k:Bacteria,p:Deinococcota,c:Deinococci,o:Deinococcales,f:Deinococcaceae,g:Deinococcus,s:Uncultured bacterium

128 OTU_3998:k:Bacteria,p:Firmicutes,c:Bacilli,o:Bacillales,f:Bacillaceae

129 OTU_3544:k:Bacteria,p:Firmicutes,c:Clostridia,o:Lachnospirales,f:Lachnospiraceae,g:Lachnospiraceae NK4A136 group

130 OTU_2568:k:Bacteria,p:Bacteroidota,c:Bacteroidia,o:Bacteroidales,f:Bacteroidaceae,g:Bacteroides,s:Bacteroides paurosaccharolyticus-JCM-15092

131 OTU_1502:k:Bacteria,p:Actinobacteriota,c:Actinobacteria,o:Corynebacteriales,f:Corynebacteriaceae,g:Corynebacterium,s:Corynebacterium pseudodiphtheriticum

132 OTU_2732:k:Bacteria,p:Bacteroidota,c:Kapabacteria,o:Kapabacteriales

133 OTU_851:k:Bacteria,p:Fusobacteriota,c:Fusobacteriia,o:Fusobacteriales,f:Leptotrichiaceae,g:Leptotrichia,s:uncultured Leptotrichia

134 OTU_1326:k:Bacteria,p:Firmicutes,c:Clostridia,o:Peptostreptococcales-Tissierellales

135 OTU_3213:k:Bacteria,p:Bacteroidota,c:Bacteroidia,o:Flavobacteriales,f:Flavobacteriaceae,g:Flavobacterium,s:Uncultured bacterium

136 OTU_3690:k:Bacteria,p:Proteobacteria,c:Gammaproteobacteria,o:Pseudomonadales,f:Moraxellaceae,g:Alkanindiges,s:uncultured bacterium

137 OTU_2113:k:Bacteria,p:Firmicutes,c:Bacilli,o:Paenibacillales,f:Paenibacillaceae,g:Cohnella,s:Uncultured Cohnella

138 OTU_3952:k:Bacteria,p:Firmicutes,c:Clostridia,o:Eubacteriales,f:Eubacteriaceae,g:Acetobacterium,s:Acetobacterium wieringae

139 OTU_2657:k:Bacteria,p:Firmicutes,c:Thermoanaerobacteria,o:Thermoanaerobacterales,f:SRB2

140 OTU_1715:k:Bacteria,p:Actinobacteriota,c:Actinobacteria,o:Propionibacteriales,f:Propionibacteriaceae

141 OTU_1699:k:Bacteria,p:Actinobacteriota,c:Thermoleophilia,o:Solirubrobacterales,f:67-14

142 OTU_2277:k:Bacteria,p:Firmicutes,c:Clostridia,o:Lachnospirales,f:Lachnospiraceae,g:Lachnospiraceae-NK4A136-group

143 OTU_3188:k:Bacteria,p:Proteobacteria,c:Gammaproteobacteria,o:Pasteurellales,f:Pasteurellaceae,g:Haemophilus,s:Haemophilus parainfluenzae

144 OTU_2522:k:Bacteria,p:Actinobacteriota,c:Thermoleophilia,o:Solirubrobacterales,f:67-14

145 OTU_1643:k:Bacteria,p:Firmicutes,c:Clostridia,o:Oscillospirales,f:Oscillospiraceae,g:Oscillospira,s:uncultured bacterium

146 OTU_3592:k:Bacteria,p:Proteobacteria,c:Gammaproteobacteria,o:Burkholderiales,f:Comamonadaceae,g:Variovorax,s:Variovorax ginsengisoli

147 OTU_1490:k:Bacteria,p:Acidobacteriota,c:Vicinamibacteria,o:Vicinamibacterales,f:uncultured

148 OTU_3167:k:Bacteria,p:Proteobacteria,c:Alphaproteobacteria,o:Sphingomonadales,f:Sphingomonadaceae,g:Sphingomonas

149 OTU_1866:k:Bacteria,p:Deinococcota,c:Deinococci,o:Deinococcales,f:Deinococcaceae,g:Deinococcus,s:Uncultured

150 OTU_2310:k:Bacteria,p:Proteobacteria,c:Gammaproteobacteria,o:Gammaproteobacteria Incertae Sedis,f:Unknown-Family,g:Candidatus Berkiella,s:Uncultured

151 OTU_3933:k:Bacteria,p:Deinococcota,c:Deinococci,o:Deinococcales,f:Deinococcaceae,g:Deinococcus,s:Deinococcus 3B1

152 OTU_4238:k:Bacteria,p:Bacteroidota,c:Bacteroidia,o:Bacteroidales,f:Prevotellaceae,g:Prevotella,s:uncultured bacterium

153 OTU_2984:k:Bacteria,p:Firmicutes,c:Bacilli,o:Lactobacillales,f:Lactobacillaceae,g:Lactobacillus

154 OTU_1520:k:Bacteria,p:Actinobacteriota,c:Actinobacteria,o:Micrococcales

155 OTU_2501:k:Bacteria,p:Proteobacteria,c:Gammaproteobacteria,o:Burkholderiales,f:Oxalobacteraceae,g:Massilia,s:Uncultured

156 OTU_1821:k:Bacteria,p:Bacteroidota,c:Bacteroidia,o:Flavobacteriales,f:Weeksellaceae,g:Chryseobacterium

157 OTU_1587:k:Bacteria,p:Proteobacteria,c:Alphaproteobacteria,o:Sphingomonadales,f:Sphingomonadaceae,g:Altererythrobacter,s:uncultured

158 OTU_1010:k:Bacteria,p:Bacteroidota,c:Bacteroidia,o:Bacteroidales,f:Prevotellaceae,g:Prevotellaceae-Ga6A1-group,s:uncultured

159 OTU_3622:k:Bacteria,p:Actinobacteriota,c:Actinobacteria,o:Propionibacteriales,f:Nocardioidaceae,g:Aeromicrobium

160 OTU_2473:k:Bacteria,p:Desulfobacterota,c:Desulfobulbia,o:Desulfobulbales,f:Desulfobulbaceae,g:Desulfobulbus,s:uncultured

161 OTU_3567:k:Bacteria,p:Proteobacteria,c:Gammaproteobacteria,o:Pasteurellales,f:Pasteurellaceae,s:Actinobacillus porcinus

162 OTU_2270:k:Bacteria,p:Bacteroidota,c:Bacteroidia,o:Bacteroidales,f:Muribaculaceae

163 OTU_2115:k:Bacteria,p:Proteobacteria,c:Gammaproteobacteria,o:Burkholderiales,f:Comamonadaceae

164 OTU_2416:k:Bacteria,p:Firmicutes,c:Bacilli,o:Lactobacillales,f:Lactobacillaceae,g:Lactobacillus

165 OTU_3598:k:Bacteria,p:Firmicutes,c:Clostridia,o:Oscillospirales,f:Ruminococcaceae,g:uncultured,s:uncultured bacterium

166 OTU_4306:k:Bacteria,p:Bacteroidota,c:Bacteroidia,o:Bacteroidales,f:Prevotellaceae,g:Prevotellaceae-NK3B31-group,s:uncultured

167 OTU_1702:k:Bacteria,p:Proteobacteria,c:Alphaproteobacteria,o:Sphingomonadales,f:Sphingomonadaceae

168 OTU_2459:k:Bacteria,p:Bacteroidota,c:Bacteroidia,o:Cytophagales,f:Spirosomaceae,g:Dyadobacter

169 OTU_2843:k:Bacteria,p:Bacteroidota,c:Bacteroidia,o:Chitinophagales,f:Saprospiraceae

170 OTU_2806:k:Bacteria,p:Firmicutes,c:Bacilli,o:Erysipelotrichales,f:Erysipelatoclostridiaceae,g:Candidatus Stoquefichus,s:Longibaculum muris

171 OTU_1015:k:Bacteria,p:Proteobacteria,c:Gammaproteobacteria,o:Burkholderiales,f:Sutterellaceae,g:Sutterella

172 OTU_975:k:Bacteria,p:Actinobacteriota,c:Actinobacteria,o:Actinomycetales,f:Actinomycetaceae,g:F0332,s:uncultured bacterium

173	OTU_2173:k:Bacteria,p:Firmicutes,c:Bacilli,o:Bacillales,f:Planococcaceae,g:Sporosarcina
174	OTU_650:k:Bacteria,p:Proteobacteria,c:Alphaproteobacteria,o:Rickettsiales,f:Rickettsiaceae,g:Rickettsia,s:Rickettsia endosymbiont
175	OTU_1429:k:Bacteria,p:Proteobacteria,c:Alphaproteobacteria,o:Rhizobiales,f:Beijerinckiaceae,g:uncultured
176	OTU_1375:k:Bacteria,p:Actinobacteriota,c:Actinobacteria,o:Micrococcales,f:Microbacteriaceae,g:Agromyces,s:Agromyces iriomotensis
177	OTU_836:k:Bacteria,p:Proteobacteria,c:Gammaproteobacteria,o:Burkholderiales,f:Neisseriaceae,g:Vitreoscilla,s:uncultured Vitreoscilla
178	OTU_1142:k:Bacteria,p:Firmicutes,c:Clostridia,o:Lachnospirales,f:Lachnospiraceae,g:Lachnospiraceae NK4A136 group,s:uncultured
179	OTU_1746:k:Bacteria,p:Verrucomicrobiota,c:Chlamydiae,o:Chlamydiales,f:Parachlamydiaceae,g:Neochlamydia
180	OTU_838:k:Bacteria,p:Firmicutes,c:Clostridia,o:Clostridiales,f:Clostridiaceae,g:Proteiniclasticum
181	OTU_1432:k:Bacteria,p:Firmicutes,c:Clostridia,o:Lachnospirales,f:Lachnospiraceae, g:Lachnospira,s:uncultured bacterium
182	OTU_1183:k:Bacteria,p:Firmicutes,c:Clostridia,o:Oscillospirales,f:Ruminococcaceae,g:Incertae Sedis,s:Uncultured bacterium
183	OTU_1878:k:Bacteria,p:Firmicutes,c:Clostridia,o:Eubacteriales,f:Anaerofustaceae,g:Anaerofustis,s:uncultured bacterium
184	OTU_1344:k:Bacteria,p:Bacteroidota,c:Bacteroidia,o:Sphingobacteriales,f:Sphingobacteriaceae,g:Sphingobacterium,s:Sphingobacterium shayense
185	OTU_1714:k:Bacteria,p:Actinobacteriota,c:Coriobacteriia,o:OPB41
186	OTU_1019:k:Bacteria,p:Bacteroidota,c:Bacteroidia,o:Flavobacteriales,f:Flavobacteriaceae,g:Capnocytophaga
187	OTU_2090:k:Bacteria,p:Bdellovibrionota,c:Bdellovibrionia,o:Bacteriovoracales,f:Bacteriovoracaceae,g:Peredibacter,s:Uncultured bacterium
188	OTU_1573:k:Bacteria,p:Firmicutes,c:Clostridia,o:Christensenellales,f:Christensenellaceae,g:uncultured,s:uncultured bacterium
189	OTU_1910:k:Bacteria,p:Bacteroidota,c:Bacteroidia,o:Bacteroidales,f:Bacteroidaceae,g:Bacteroides
190	OTU_1480:k:Bacteria,p:Proteobacteria,c:Alphaproteobacteria,o:Acetobacterales,f:Acetobacteraceae,g:uncultured,s:uncultured bacterium
191	OTU_999:k:Bacteria,p:Actinobacteriota,c:Actinobacteria
192	OTU_1634:k:Bacteria,p:Bacteroidota,c:Bacteroidia,o:Bacteroidales,f:Rikenellaceae,g:Alistipes,s:uncultured
193	OTU_1434:k:Bacteria,p:Verrucomicrobiota,c:Verrucomicrobiae,o:Verrucomicrobiales,f:Verrucomicrobiaceae,g:uncultured,s:uncultured verrucomicrobium
194	OTU_1445:k:Bacteria,p:Firmicutes,c:Clostridia,o:Christensenellales,f:Christensenellaceae,g:uncultured,s:uncultured bacterium
195	OTU_2988:k:Bacteria,p:Actinobacteriota,c:Actinobacteria,o:Propionibacteriales,f:Propionibacteriaceae
196	OTU_2643:k:Bacteria,p:Campilobacterota,c:Campylobacteria,o:Campylobacterales,f:Campylobacteraceae,g:Campylobacter,s:Campylobacter gracilis
197	OTU_4173:k:Bacteria,p:Bacteroidota,c:Bacteroidia,o:Flavobacteriales,f:Flavobacteriaceae,g:Flavobacterium,s:Flavobacterium V22012
198	OTU_4141:k:Bacteria,p:Bacteroidota,c:Bacteroidia,o:Bacteroidales,f:Dysgonomonadaceae,g:Dysgonomonas
199	OTU_3993:k:Bacteria,p:Bacteroidota,c:Bacteroidia,o:Bacteroidales,f:Marinifilaceae,g:Butyricimonas
200	OTU_1707:k:Bacteria,p:Deinococcota,c:Deinococci,o:Deinococcales,f:Deinococcaceae,g:Deinococcus,s:Deinococcus geothermalis
201	OTU_3597:k:Bacteria,p:Firmicutes,c:Clostridia,o:Lachnospirales,f:Lachnospiraceae,g:Lachnospira,s:uncultured bacterium
202	OTU_4290:k:Bacteria,p:Bacteroidota,c:Bacteroidia,o:Bacteroidales,f:Prevotellaceae,g:Prevotellaceae UCG-003

Keynote: k: kingdom, p: phylum, c:class, o: order, f: family, g: genus, s: species

Supplementary Table S8. Unique 216 OTUs in stomach samples, contributing to variance in the overall sequences.

No.	OTUs descriptions
1	OTU_2550:k:Bacteria,p:Bacteroidota,c:Bacteroidia,o:Bacteroidales,f:Prevotellaceae,g:Prevotella,s:uncultured bacterium
2	OTU_3023:k:Bacteria,p:Actinobacteriota,c:Actinobacteria,o:Pseudonocardiales,f:Pseudonocardiaceae
3	OTU_1667:k:Bacteria,p:Actinobacteriota,c:Coriobacteriia,o:OPB41
4	OTU_2365:k:Bacteria,p:Actinobacteriota,c:Actinobacteria,o:Frankiales,f:Nakamurellaceae,g:Nakamurella,s:Uncultured
5	OTU_905:k:Bacteria,p:Firmicutes,c:Clostridia,o:Oscillospirales,f:UCG-010
6	OTU_1955:k:Bacteria,p:Bacteroidota,c:Bacteroidia,o:Flavobacteriales,f:Flavobacteriaceae,g:Flavobacterium
7	OTU_4212:k:Bacteria,p:Chloroflexi,c:Chloroflexia,o:Chloroflexales,f:Roseiflexaceae
8	OTU_3201:k:Bacteria,p:Proteobacteria,c:Alphaproteobacteria,o:Tistrellales,f:Geminicoccaceae,g:Candidatus Alysiosphaera
9	OTU_2633:k:Bacteria,p:Bacteroidota,c:Bacteroidia,o:Flavobacteriales,f:Flavobacteriaceae,g:Flavobacterium
10	OTU_1991:k:Bacteria,p:Proteobacteria,c:Gammaproteobacteria,o:Aeromonadales,f:Aeromonadaceae,g:Aeromonas
11	OTU_2936:k:Bacteria,p:Bacteroidota,c:Bacteroidia,o:Chitinophagales,f:Chitinophagaceae
12	OTU_4305:k:Bacteria,p:Chloroflexi,c:JG30-KF-CM66
13	OTU_3565:k:Bacteria,p:Firmicutes,c:Clostridia,o:Monoglobales,f:Monoglobaceae,g:Monoglobus,s:uncultured bacterium
14	OTU_4050:k:Bacteria,p:Proteobacteria,c:Alphaproteobacteria,o:Ferrovibrionales,g:Taonella,s:Taonella mepensis
15	OTU_3097:k:Bacteria,p:Bacteroidota,c:Bacteroidia,o:Bacteroidales,f:Prevotellaceae,g:Prevotella
16	OTU_1898:k:Bacteria,p:Actinobacteriota,c:Actinobacteria,o:Propionibacteriales,f:Nocardioidaceae,g:Nocardioides,s:uncultured bacterium
17	OTU_2298:k:Bacteria,p:Firmicutes,c:Bacilli,o:Lactobacillales,f:Lactobacillaceae,g:Lactobacillus,s:Uncultured bacterium
18	OTU_1970:k:Bacteria,p:Chloroflexi,c:Chloroflexia,o:Thermomicrobiales,f:JG30-KF-CM45
19	OTU_2521:k:Bacteria,p:Actinobacteriota,c:Thermoleophilia,o:Solirubrobacterales,f:67-14
20	OTU_3850:k:Bacteria,p:Proteobacteria,c:Alphaproteobacteria,o:Rhodobacterales,f:Rhodobacteraceae
21	OTU_4047:k:Bacteria,p:Actinobacteriota,c:Actinobacteria,o:Corynebacteriales,f:Nocardiaceae,g:Rhodococcus,s:Rhodococcus corynebacterioides
22	OTU_1462:k:Bacteria,p:Bacteroidota,c:Bacteroidia,o:Bacteroidales,f:Rikenellaceae,g:Alistipes
23	OTU_3564:k:Bacteria,p:Firmicutes,c:Bacilli,o:Lactobacillales,f:Lactobacillaceae,g:Lactobacillus,s:Uncultured bacterium
24	OTU_3628:k:Bacteria,p:Firmicutes,c:Bacilli,o:Thermoactinomycetales,f:Thermoactinomycetaceae,g:Kroppenstedtia
25	OTU_2982:k:Bacteria,p:Bacteroidota,c:Bacteroidia,o:Bacteroidales,f:Muribaculaceae

26 OTU_3573:k:Bacteria,p:Firmicutes,c:Bacilli

27 OTU_3096:k:Bacteria,p:Actinobacteriota,c:Actinobacteria,o:Micrococcales

28 OTU_1935:k:Bacteria,p:Actinobacteriota,c:Actinobacteria,o:Actinomycetales,f:Actinomycetaceae,g:Actinomyces,s:Actinomyces gerencseriae

29 OTU_3140:k:Bacteria

30 OTU_4115:k:Bacteria,p:Proteobacteria,c:Alphaproteobacteria,o:Rhodospirillales,f:uncultured

31 OTU_3893:k:Bacteria,p:Firmicutes,c:Clostridia,o:Oscillospirales,f:[Eubacterium]-coprostanoligenes group

32 OTU_1831:k:Bacteria,p:Firmicutes,c:Clostridia,o:Lachnospirales,f:Lachnospiraceae,g:[Eubacterium]-eligens group

33 OTU_4219:k:Bacteria,p:Firmicutes,c:Clostridia,o:Oscillospirales,f:Oscillospiraceae,g:uncultured,s:uncultured bacterium

34 OTU_3392:k:Bacteria,p:Firmicutes,c:Clostridia,o:Lachnospirales,f:Lachnospiraceae

35 OTU_1849:k:Bacteria,p:Firmicutes,c:Negativicutes,o:Veillonellales-Selenomonadales,f:Sporomusaceae,g:Anaeromusa-Anaeroarcus,s:uncultured bacterium

36 OTU_2223:k:Bacteria,p:Bacteroidota,c:Bacteroidia,o:Bacteroidales,f:Rikenellaceae

37 OTU_892:k:Bacteria,p:Deinococcota,c:Deinococci,o:Deinococcales,f:Deinococcaceae,g:Deinococcus,s:Deinococcus J16OP19

38 OTU_4042:k:Bacteria,p:Bacteroidota,c:Bacteroidia,o:Bacteroidales,f:Muribaculaceae

39 OTU_1438:k:Bacteria,p:Firmicutes,c:Clostridia,o:Monoglobales,f:Monoglobaceae,g:Monoglobus,s:uncultured

40 OTU_2535:k:Bacteria,p:Firmicutes,c:Clostridia,o:Clostridia UCG-014

41 OTU_1839:k:Bacteria,p:Firmicutes,c:Clostridia,o:Lachnospirales,f:Lachnospiraceae,g:Lachnospira

42 OTU_1563:k:Bacteria,p:Firmicutes,c:Clostridia,o:Lachnospirales,f:Lachnospiraceae,g:Oribacterium,s:uncultured bacterium

43 OTU_4008:k:Bacteria,p:Firmicutes,c:Clostridia,o:Christensenellales,f:Christensenellaceae,g:Christensenellaceae-R-7-group,s:uncultured bacterium

44 OTU_2553:k:Bacteria,p:Chloroflexi,c:Dehalococcoidia,o:Dehalococcoidales,f:Dehalococcoidaceae,g:Dehalococcoides,s:Dehalococcoides mccartyi

45 OTU_4000:k:Bacteria,p:Bacteroidota,c:Bacteroidia,o:Bacteroidales,f:Prevotellaceae,g:Alloprevotella,s:uncultured bacterium

46 OTU_4421:k:Bacteria,p:Actinobacteriota,c:Actinobacteria,o:Micrococcales,f:Micrococcaceae

47 OTU_1886:k:Bacteria,p:Firmicutes,c:Clostridia,o:Oscillospirales,f:Butyricicoccaceae,g:UCG-009,s:uncultured bacterium

48 OTU_3975:k:Bacteria,p:Firmicutes,c:Bacilli,o:Lactobacillales,f:Lactobacillaceae,g:Lactobacillus,s:Lactobacillus dextrinicus

49 OTU_3327:k:Bacteria,p:Proteobacteria,c:Alphaproteobacteria,o:Rhodospirillales,f:uncultured

50 OTU_1396:k:Bacteria,p:Firmicutes,c:Clostridia,o:Clostridiales,f:Clostridiaceae,g:Clostridium-sensu-stricto-7,s:uncultured

51 OTU_3956:k:Bacteria,p:Firmicutes,c:Clostridia,o:Oscillospirales,f:Ruminococcaceae,g:Anaerofilum

52 OTU_2467:k:Bacteria,p:Firmicutes,c:Clostridia,o:Lachnospirales,f:Lachnospiraceae

53 OTU_3963:k:Bacteria,p:Actinobacteriota,c:Coriobacteriia,o:Coriobacteriales,f:Eggerthellaceae

54 OTU_4504:k:Bacteria,p:Actinobacteriota,c:Actinobacteria,o:Actinomycetales,f:Actinomycetaceae,g:Actinomyces

55 OTU_2021:k:Bacteria,p:Firmicutes,c:Bacilli,o:Lactobacillales

OTU_3404:k:Bacteria,p:Bacteroidota,c:Bacteroidia,o:Bacteroidales,f:Muribaculaceae

OTU_2931:k:Bacteria,p:Proteobacteria,c:Gammaproteobacteria,o:Cardiobacteriales,f:Cardiobacteriaceae,g:Cardiobacterium

OTU_3844:k:Bacteria,p:Bacteroidota,c:Bacteroidia,o:Bacteroidales

OTU_4296:k:Bacteria

OTU_3978:k:Bacteria,p:Cyanobacteria,c:Cyanobacteriia,o:Phormidesmiales,f:Phormidesmiaceae,g:Phormidesmis ANT.LACV5.1

OTU_4310:k:Bacteria,p:Firmicutes,c:Clostridia,o:Clostridiales,f:Clostridiaceae,g:Youngiibacter,s:Youngiibacter multivorans

OTU_945:k:Bacteria,p:Bacteroidota,c:Bacteroidia,o:Flavobacteriales,f:Flavobacteriaceae,g:Capnocytophaga

OTU_4442:k:Bacteria,p:Firmicutes,c:Clostridia,o:Lachnospirales,f:Lachnospiraceae

OTU_4402:k:Bacteria,p:Firmicutes,c:Clostridia,o:Lachnospirales,f:Lachnospiraceae

OTU_2328:k:Bacteria,p:Firmicutes,c:Clostridia,o:Lachnospirales,f:Lachnospiraceae,g:Blautia,s:uncultured bacterium

OTU_2248:k:Bacteria,p:Firmicutes,c:Clostridia,o:Oscillospirales,f:UCG-010

OTU_1700:k:Bacteria

OTU_2586:k:Bacteria,p:Firmicutes,c:Clostridia,o:Clostridiales,f:Clostridiaceae,g:Clostridium-sensu-stricto-8,s:Unculture

OTU_1926:k:Bacteria,p:Desulfobacterota,c:Desulfobulbia,o:Desulfobulbales,f:Desulfobulbaceae,g:uncultured,s:uncultured bacterium

OTU_1815:k:Bacteria,p:Cyanobacteria,c:Cyanobacteriia,o:Synechococcales,f:Cyanobiaceae,g:Cyanobium PCC-6307

OTU_2186:k:Bacteria,p:Acidobacteriota,c:Vicinamibacteria,o:Vicinamibacterales,f:Vicinamibacteraceae

OTU_3812:k:Bacteria,p:Acidobacteriota

OTU_1950:k:Bacteria,p:Proteobacteria,c:Gammaproteobacteria,o:Burkholderiales

OTU_2519:k:Bacteria,p:Actinobacteriota,c:Coriobacteriia,o:Coriobacteriales,f:Eggerthellaceae

OTU_2042:k:Bacteria,p:Nitrospirota,c:Thermodesulfovibrionia,o:uncultured

OTU_3319:k:Bacteria

OTU_2262:k:Bacteria,p:Chloroflexi,c:Anaerolineae,o:ADurb.Bin180

OTU_4069:k:Bacteria

OTU_1475:k:Bacteria,p:Firmicutes,c:Clostridia,o:Lachnospirales,f:Defluviitaleaceae,g:Defluviitaleaceae UCG-011,s:uncultured bacterium

OTU_4404:k:Bacteria,p:Bacteroidota,c:Bacteroidia,o:Bacteroidales,f:Muribaculaceae

OTU_4150:k:Bacteria,p:Firmicutes,c:Clostridia,o:Lachnospirales,f:Lachnospiraceae

OTU_1427:k:Bacteria,p:Firmicutes,c:Clostridia

OTU_4092:k:Bacteria,p:Firmicutes,c:Bacilli

OTU_4093:k:Bacteria

OTU_2547:k:Bacteria,p:Firmicutes,c:Clostridia,o:Christensenellales,f:Christensenellaceae,g:Christensenella,s:Christensenella minuta

OTU_4007:k:Bacteria

87 OTU_1820:k:Bacteria,p:Firmicutes,c:Clostridia,o:Peptostreptococcales-Tissierellales,f:Anaerovoracaceae,g:[Eubacterium]-nodatum

88 OTU_4509:k:Bacteria,p:Firmicutes,c:Clostridia,o:Oscillospirales,f:Oscillospiraceae

89 OTU_4005:k:Bacteria

90 OTU_2228:k:Bacteria,p:Bacteroidota,c:Bacteroidia,o:Bacteroidales,f:Muribaculaceae

91 OTU_1282:k:Bacteria,p:Firmicutes,c:Bacilli,o:Erysipelotrichales,f:Erysipelotrichaceae,g:Clostridium innocuum

92 OTU_3006:k:Bacteria,p:Actinobacteriota,c:Actinobacteria

93 OTU_1524:k:Bacteria,p:Bacteroidota,c:Bacteroidia,o:Bacteroidales,f:Muribaculaceae

94 OTU_3992:k:Bacteria,p:Firmicutes,c:Clostridia,o:Lachnospirales,f:Lachnospiraceae

95 OTU_3466:k:Bacteria,p:Firmicutes,c:Clostridia,o:Lachnospirales,f:Lachnospiraceae

96 OTU_2029:k:Bacteria,p:Firmicutes,c:Bacilli,o:Erysipelotrichales,f:Erysipelatoclostridiaceae

97 OTU_3008:k:Bacteria,p:Firmicutes

98 OTU_4010:k:Bacteria,p:Firmicutes,c:Clostridia,o:Lachnospirales,f:Lachnospiraceae

99 OTU_2374:k:Bacteria,p:Firmicutes,c:Clostridia,o:Lachnospirales,f:Lachnospiraceae

100 OTU_1860:k:Bacteria,p:Firmicutes,c:Clostridia,o:Oscillospirales,f:Oscillospiraceae,g:NK4A214 group

101 OTU_3824:k:Bacteria,p:Firmicutes,c:Clostridia,o:Peptostreptococcales-Tissierellales,f:Anaerovoracaceae

102 OTU_3852:k:Bacteria,p:Firmicutes,c:Clostridia,o:Lachnospirales,f:Lachnospiraceae

103 OTU_4480:k:Bacteria,p:Firmicutes,c:Clostridia,o:Oscillospirales,f:Ruminococcaceae,g:uncultured,s:uncultured organism

104 OTU_3073:k:Bacteria,p:Bacteroidota,c:Bacteroidia,o:Bacteroidales,f:Muribaculaceae

105 OTU_3003:k:Bacteria,p:Bacteroidota,c:Bacteroidia,o:Bacteroidales

106 OTU_4454:k:Bacteria,p:Firmicutes,c:Clostridia,o:Lachnospirales,f:Lachnospiraceae

107 OTU_4453:k:Bacteria,p:Bacteroidota,c:Bacteroidia,o:Bacteroidales,f:Muribaculaceae

108 OTU_2998:k:Bacteria,p:Firmicutes,c:Clostridia,o:Lachnospirales,f:Lachnospiraceae,g:Howardella

109 OTU_4015:k:Bacteria,p:Firmicutes,c:Clostridia,o:Lachnospirales,f:Lachnospiraceae

110 OTU_2495:k:Bacteria

111 OTU_3980:k:Bacteria,p:Firmicutes,c:Bacilli,o:Lactobacillales,f:Lactobacillaceae,g:Lactobacillus,s:Uncultured Lactobacillus

112 OTU_2558:k:Bacteria,p:Firmicutes,c:Clostridia,o:Oscillospirales,f:Ethanoligenenaceae,g:Acetanaerobacterium,s:uncultured bacterium

113 OTU_1808:k:Bacteria,p:Actinobacteriota,c:Actinobacteria,o:Corynebacteriales,f:Corynebacteriaceae,g:Corynebacterium,s:Uncultured bacterium

114 OTU_2575:k:Bacteria,p:Firmicutes,c:Clostridia,o:Lachnospirales,f:Lachnospiraceae

115 OTU_3090:k:Bacteria,p:Firmicutes,c:Bacilli,o:Lactobacillales,f:Lactobacillaceae,g:Lactobacillus,s:Uncultured bacterium

116 OTU_4514:k:Bacteria,p:Firmicutes

117 OTU_3088:k:Bacteria,p:Firmicutes,c:Clostridia,o:Oscillospirales,g:Hydrogenoanaerobacterium,s:uncultured Clostridium

118 OTU_3965:k:Bacteria,p:Bacteroidota,c:Bacteroidia,o:Bacteroidales,f:Muribaculaceae
119 OTU_4289:k:Bacteria,p:Bacteroidota,c:Bacteroidia,o:Bacteroidales,f:Muribaculaceae
120 OTU_675:k:Bacteria,p:Firmicutes,c:Clostridia,o:Lachnospirales,f:Lachnospiraceae
121 OTU_4218:k:Bacteria
122 OTU_4329:k:Bacteria,p:Firmicutes,c:Clostridia,o:Lachnospirales,f:Lachnospiraceae,g:Blautia,s:uncultured bacterium
123 OTU_4274:k:Bacteria,p:Bacteroidota,c:Bacteroidia,o:Bacteroidales,f:Muribaculaceae
124 OTU_3083:k:Bacteria,p:Firmicutes,c:Clostridia,o:Lachnospirales,f:Lachnospiraceae
125 OTU_4324:k:Bacteria
126 OTU_3077:k:Bacteria
127 OTU_3078:k:Bacteria,p:Firmicutes,c:Clostridia,o:Lachnospirales,f:Lachnospiraceae
128 OTU_2909:k:Bacteria,p:Bacteroidota,c:Bacteroidia,o:Bacteroidales,f:Bacteroidaceae,g:Bacteroides
129 OTU_3767:k:Bacteria,p:Firmicutes,c:Clostridia,o:Eubacteriales,f:Anaerofustaceae,g:Anaerofustis
130 OTU_4207:k:Bacteria,p:Bacteroidota,c:Bacteroidia,o:Bacteroidales,f:Rikenellaceae,g:Rikenellaceae RC9 gut group
131 OTU_2676:k:Bacteria,p:Firmicutes
132 OTU_2714:k:Bacteria,p:Firmicutes,c:Bacilli,o:Lactobacillales,f:Streptococcaceae,g:Streptococcus,s:Uncultured Streptococcus
133 OTU_3045:k:Bacteria,p:Firmicutes,c:Bacilli,o:Lactobacillales,f:Lactobacillaceae,g:Lactobacillus,s:Lactobacillus mucosae
134 OTU_3969:k:Bacteria,p:Firmicutes,c:Clostridia,o:Lachnospirales,f:Lachnospiraceae
135 OTU_3973:k:Bacteria,p:Firmicutes
136 OTU_2979:k:Bacteria,p:Bacteroidota,c:Bacteroidia,o:Bacteroidales,f:Muribaculaceae
137 OTU_4354:k:Bacteria,p:Firmicutes,c:Clostridia,o:Oscillospirales,f:Oscillospiraceae,g:Papillibacter,s:uncultured bacterium
138 OTU_2992:k:Bacteria,p:Firmicutes
139 OTU_1509:k:Bacteria,p:Firmicutes,c:Clostridia,o:Lachnospirales,f:Lachnospiraceae,g:Lachnospiraceae UCG-001
140 OTU_2593:k:Bacteria,p:Firmicutes,c:Bacilli
141 OTU_3826:k:Bacteria,p:Firmicutes,c:Clostridia
142 OTU_2967:k:Bacteria,p:Firmicutes
143 OTU_4459:k:Bacteria,p:Firmicutes,c:Clostridia
144 OTU_2978:k:Bacteria
145 OTU_3818:k:Bacteria,p:Firmicutes,c:Clostridia,o:Lachnospirales,f:Lachnospiraceae
146 OTU_4077:k:Bacteria,p:Firmicutes
147 OTU_3808:k:Bacteria,p:Firmicutes,c:Clostridia,o:Lachnospirales,f:Lachnospiraceae,g:Eubacterium-xylanophilum,s:uncultured bacterium
148 OTU_3782:k:Bacteria,p:Firmicutes,c:Clostridia,o:Lachnospirales,f:Lachnospiraceae

OTU_3024:k:Bacteria,p:Firmicutes,c:Clostridia,o:Oscillospirales,f:Ruminococcaceae
OTU_3036:k:Bacteria,p:Firmicutes,c:Clostridia,o:Lachnospirales,f:Lachnospiraceae
OTU_4111:k:Bacteria,p:Firmicutes
OTU_3833:k:Bacteria
OTU_716:k:Bacteria,p:Actinobacteriota,c:Actinobacteria,o:Micrococcales
OTU_978:k:Bacteria,p:Bacteroidota,c:Bacteroidia,o:Sphingobacteriales,f:env.OPS 17
OTU_1691:k:Bacteria,p:Proteobacteria,c:Gammaproteobacteria,o:Enterobacterales,f:Morganellaceae,g:Morganella
OTU_625:k:Bacteria,p:Proteobacteria,c:Alphaproteobacteria,o:Caulobacterales,f:Caulobacteraceae,g:Brevundimonas
OTU_1738:k:Bacteria,p:Proteobacteria,c:Gammaproteobacteria,o:Burkholderiales,f:SC-I-84
OTU_1555:k:Bacteria,p:Proteobacteria,c:Gammaproteobacteria,o:Pasteurellales,f:Pasteurellaceae,g:Aggregatibacter,s:Aggregatibacter segnis
OTU_1126:k:Bacteria,p:Firmicutes,c:Bacilli,o:Lactobacillales,f:Carnobacteriaceae
OTU_1896:k:Bacteria,p:Firmicutes,c:Clostridia,o:Peptostreptococcales-Tissierellales,g:Sporanaerobacter,s:Sporanaerobacter acetigenes
OTU_2656:k:Bacteria,p:Proteobacteria,c:Gammaproteobacteria,o:Burkholderiales,f:Neisseriaceae,g:Neisseria,s:Neisseria zaloph
OTU_1466:k:Bacteria,p:Proteobacteria,c:Gammaproteobacteria,o:Burkholderiales,f:Comamonadaceae,g:Methylibium,s:Methylibium petroleiphilum
OTU_3835:k:Bacteria,p:Actinobacteriota,c:Actinobacteria,o:Corynebacteriales,f:Corynebacteriaceae,g:Corynebacterium,s:Uncultured bacterium
OTU_1759:k:Bacteria,p:Desulfobacterota,c:Desulfuromonadia
OTU_2584:k:Bacteria,p:Deinococcota,c:Deinococci,o:Deinococcales,f:Deinococcaceae,g:Deinococcus
OTU_3719:k:Bacteria,p:Firmicutes,c:Clostridia,o:Lachnospirales,f:Lachnospiraceae
OTU_1032:k:Bacteria,p:Firmicutes,c:Clostridia,o:Oscillospirales,f:UCG-010
OTU_2112:k:Bacteria,p:Firmicutes,c:Clostridia,o:Monoglobales,f:Monoglobaceae,g:Monoglobus,s:Uncultured
OTU_1118:k:Bacteria,p:Firmicutes,c:Clostridia,o:Lachnospirales,f:Lachnospiraceae,g:uncultured,s:uncultured bacterium
OTU_3131:k:Bacteria,p:Firmicutes,c:Clostridia,o:Lachnospirales,f:Lachnospiraceae,g:uncultured,s:uncultured bacterium
OTU_1832:k:Bacteria,p:Firmicutes,c:Clostridia,o:Clostridia vadinBB60 group
OTU_3703:k:Bacteria,p:Firmicutes,c:Clostridia,o:Lachnospirales,f:Lachnospiraceae
OTU_1455:k:Bacteria,p:Firmicutes,c:Clostridia,o:Lachnospirales,f:Lachnospiraceae,g:uncultured,s:uncultured bacterium
OTU_3673:k:Bacteria,p:Actinobacteriota,c:Actinobacteria,o:Micrococcales,f:Dermatophilaceae,g:uncultured,s:uncultured actinobacterium
OTU_2479:k:Bacteria,p:Firmicutes,c:Clostridia,o:Peptostreptococcales-Tissierellales,g:Tissierella,s:uncultured bacterium
OTU_3105:k:Bacteria,p:Firmicutes,c:Clostridia,o:Oscillospirales,f:Butyricicoccaceae,g:UCG-009,s:uncultured bacterium
OTU_4080:k:Bacteria,p:Actinobacteriota,c:Thermoleophilia,o:Solirubrobacterales,f:Solirubrobacteraceae,g:Patulibacter
OTU_3114:k:Bacteria,p:Cyanobacteria,c:Cyanobacteriia,o:Cyanobacteriales,f:Nostocaceae,g:uncultured,s:uncultured cyanobacterium
OTU_1630:k:Bacteria,p:Verrucomicrobiota,c:Lentisphaeria,o:Victivallales,f:Victivallaceae

OTU_1911:k:Bacteria,p:Firmicutes,c:Clostridia,o:Oscillospirales,f:Ruminococcaceae,g:Incertae Sedis,s:Uncultured bacterium

OTU_3714:k:Bacteria,p:Firmicutes,c:Bacilli,o:Lactobacillales,f:Streptococcaceae,g:Lactococcus

OTU_2121:k:Bacteria,p:Firmicutes,c:Clostridia,o:Clostridia vadinBB60 group

OTU_4233:k:Bacteria,p:Firmicutes,c:Clostridia,o:Lachnospirales,f:Lachnospiraceae

OTU_2507:k:Bacteria,p:Proteobacteria,c:Alphaproteobacteria,o:Rhizobiales,f:Xanthobacteraceae

OTU_4176:k:Bacteria,p:Bacteroidota,c:Bacteroidia,o:Bacteroidales,f:Bacteroidaceae,g:Bacteroides

OTU_3579:k:Bacteria,p:Firmicutes,c:Clostridia,o:Lachnospirales,f:Lachnospiraceae,g:Eisenbergiella,s:uncultured

OTU_3580:k:Bacteria,p:Firmicutes,c:Clostridia,o:Lachnospirales,f:Lachnospiraceae,g:Blautia,s:uncultured

OTU_1751:k:Bacteria,p:Bacteroidota,c:Bacteroidia,o:Bacteroidales,f:Bacteroidaceae,g:Bacteroides

OTU_3881:k:Bacteria,p:Proteobacteria,c:Alphaproteobacteria,o:Sphingomonadales,f:Sphingomonadaceae

OTU_4157:k:Bacteria,p:Firmicutes,c:Clostridia,o:Lachnospirales,f:Lachnospiraceae

OTU_2743:k:Bacteria,p:Firmicutes,c:Clostridia,o:Peptostreptococcales-Tissierellales

OTU_2303:k:Bacteria,p:Firmicutes,c:Bacilli,o:Lactobacillales,f:Lactobacillaceae,g:Lactobacillus,s:uncultured bacterium

OTU_3676:k:Bacteria,p:Actinobacteriota,c:Actinobacteria,o:Propionibacteriales,f:Nocardioidaceae,g:Nocardioides,s:uncultured

OTU_3675:k:Bacteria,p:Chloroflexi,c:Chloroflexia,o:Thermomicrobiales,f:JG30-KF-CM45

OTU_2289:k:Bacteria,p:Firmicutes,c:Bacilli,o:Erysipelotrichales,f:Erysipelotrichaceae,g:uncultured,s:uncultured bacterium

OTU_3193:k:Bacteria,p:Firmicutes,c:Clostridia,o:Christensenellales,f:Christensenellaceae,g:Christensenellaceae-R-7-group

OTU_3806:k:Bacteria,p:Firmicutes,c:Clostridia,o:Oscillospirales,f:Oscillospiraceae

OTU_3702:k:Bacteria,p:Firmicutes,c:Clostridia,o:Oscillospirales,f:Ruminococcaceae,g:uncultured,s:uncultured bacterium

OTU_2078:k:Bacteria,p:Firmicutes,c:Bacilli,o:Lactobacillales,f:Leuconostocaceae,g:Weissella,s:Weissella minor

OTU_2964:k:Bacteria,p:Firmicutes,c:Bacilli,o:Paenibacillales,f:Paenibacillaceae,g:Paenibacillus,s:Uncultured bacterium

OTU_3439:k:Bacteria,p:Proteobacteria,c:Gammaproteobacteria,o:Run-SP154

OTU_4198:k:Bacteria,p:Firmicutes

OTU_4295:k:Bacteria,p:Proteobacteria,c:Gammaproteobacteria,o:Burkholderiales,f:Burkholderiaceae,g:uncultured

OTU_2513:k:Bacteria,p:Firmicutes,c:Clostridia,o:Peptostreptococcales-Tissierellales,f:Anaerovoracaceae,g:Eubacterium,s:Eubacterium pyruvativorans

OTU_3428:k:Bacteria,p:Firmicutes,c:Clostridia,o:Peptostreptococcales-Tissierellales,g:Tepidimicrobium,s:uncultured bacterium

OTU_1403:k:Bacteria,p:Firmicutes,c:Bacilli,o:RF39

OTU_1810:k:Bacteria,p:Chloroflexi,c:Anaerolineae,o:SBR1031

OTU_2510:k:Bacteria,p:Firmicutes,c:Clostridia,o:Oscillospirales,f:Oscillospiraceae,g:Intestinimonas,s:Intestinimonas timonensis

OTU_2972:k:Bacteria,p:Firmicutes,c:Clostridia,o:Oscillospirales,f:Oscillospiraceae,g:uncultured,s:uncultured bacterium

OTU_3798:k:Bacteria,p:Firmicutes

211	OTU_3801:k:Bacteria,p:Firmicutes,c:Clostridia,o:Lachnospirales,f:Lachnospiraceae,g:Tyzzerella
212	OTU_3827:k:Bacteria,p:Firmicutes,c:Bacilli,o:Lactobacillales,f:Lactobacillaceae,g:Lactobacillus,s:Uncultured
213	OTU_3497:k:Bacteria,p:Firmicutes,c:Bacilli,o:Lactobacillales,f:Lactobacillaceae,g:Lactobacillus,s:Ligilactobacillus murinus
214	OTU_3076:k:Bacteria,p:Firmicutes,c:Clostridia,o:Lachnospirales,f:Lachnospiraceae
215	OTU_4055:k:Bacteria,p:Acidobacteriota,c:Blastocatellia,o:11-24
216	OTU_3049:k:Bacteria,p:Deinococcota,c:Deinococci,o:Deinococcales,f:Deinococcaceae,g:Deinococcus

Supplementary Table S9. Unique 48 OTUs in gut samples, contributing to variance in the overall sequences.

No.	OTUs descriptions
1	OTU_2233:k:Bacteria,p:Proteobacteria,c:Gammaproteobacteria,o:Orbales,f:Orbaceae,g:Frischella,s:Frischella perrara
2	OTU_3865:k:Bacteria,p:Campilobacterota,c:Campylobacteria,o:Campylobacterales,f:Helicobacteraceae,g:Helicobacter,s:Uncultured
3	OTU_2360:k:Bacteria,p:Proteobacteria,c:Alphaproteobacteria,o:Acetobacterales,f:Acetobacteraceae,g:Bombella
4	OTU_220:k:Bacteria,p:Proteobacteria,c:Gammaproteobacteria,o:Burkholderiales,f:Burkholderiaceae,g:Cupriavidus
5	OTU_2185:k:Bacteria,p:Firmicutes,c:Bacilli,o:Lactobacillales,f:Lactobacillaceae,g:Lactobacillus,s:Lactobacillus apis
6	OTU_3038:k:Bacteria,p:Firmicutes
7	OTU_4118:k:Bacteria,p:Firmicutes,c:Bacilli,o:Lactobacillales,f:Lactobacillaceae,g:Lactobacillus,s:Apilactobacillus kunkeei
8	OTU_1549:k:Bacteria,p:Proteobacteria,c:Gammaproteobacteria,o:Orbales,f:Orbaceae,g:Gilliamella,s:gamma proteobacterium
9	OTU_3977:k:Bacteria,p:Firmicutes,c:Bacilli
10	OTU_2954:k:Bacteria,p:Proteobacteria,c:Gammaproteobacteria,o:Burkholderiales
11	OTU_770:k:Bacteria,p:Proteobacteria,c:Gammaproteobacteria,o:Burkholderiales,f:Oxalobacteraceae,g:Herbaspirillum
12	OTU_4193:k:Bacteria,p:Patescibacteria,c:Saccharimonadia,o:Saccharimonadales,f:Saccharimonadaceae,g:TM7a,s:uncultured
13	OTU_3586:k:Bacteria,p:Firmicutes,c:Bacilli,o:RF39
14	OTU_4025:k:Bacteria,p:Proteobacteria,c:Gammaproteobacteria,o:Burkholderiales,f:Neisseriaceae,g:Snodgrassella,s:Snodgrassella alvi
15	OTU_4059:k:Bacteria,p:Firmicutes,c:Clostridia,o:Oscillospirales,f:Ruminococcaceae,g:Faecalibacterium,s:uncultured
16	OTU_2624:k:Bacteria
17	OTU_4351:k:Bacteria,p:Firmicutes,c:Clostridia,o:Oscillospirales,f:[Eubacterium]-coprostanoligenes group
18	OTU_3186:k:Bacteria
19	OTU_2480:k:Bacteria
20	OTU_4194:k:Bacteria,p:Firmicutes,c:Bacilli,o:Erysipelotrichales,f:Erysipelotrichaceae,g:Turicibacter,s:Uncultured bacterium
21	OTU_4327:k:Bacteria
22	OTU_4241:k:Bacteria,p:Firmicutes,c:Clostridia,o:Peptostreptococcales-Tissierellales,f:Peptostreptococcaceae,g:Romboutsia,s:uncultured
23	OTU_4319:k:Bacteria,p:Actinobacteriota,c:Actinobacteria,o:Actinomycetales,f:Actinomycetaceae,g:Actinomyces
24	OTU_3807:k:Bacteria,p:Proteobacteria,c:Gammaproteobacteria,o:Burkholderiales
25	OTU_1519:k:Bacteria,p:Bacteroidota,c:Bacteroidia,o:Chitinophagales,f:Chitinophagaceae

26	OTU_3793:k:Bacteria,p:Bacteroidota,c:Bacteroidia,o:Flavobacteriales,f:Weeksellaceae,g:Apibacter,s:uncultured Bacteroidetes
27	OTU_3701:k:Bacteria,p:Proteobacteria,c:Gammaproteobacteria,o:Burkholderiales,f:Burkholderiaceae,g:Ralstonia,s:Ralstonia solanacearum
28	OTU_2805:k:Bacteria,p:Firmicutes,c:Bacilli,o:Erysipelotrichales,f:Erysipelotrichaceae,g:Ileibacterium,s:Ileibacterium valens
29	OTU_1972:k:Bacteria,p:Verrucomicrobiota,c:Verrucomicrobiae,o:Verrucomicrobiales,f:Akkermansiaceae,g:Akkermansia,s:uncultured
30	OTU_1773:k:Bacteria,p:Chloroflexi,c:Anaerolineae,o:Caldilineales,f:Caldilineaceae,g:uncultured,s:uncultured bacterium
31	OTU_1206:k:Bacteria,p:Firmicutes,c:Clostridia,o:Clostridia_vadinBB60_group
32	OTU_3734:k:Bacteria,p:Firmicutes,c:Bacilli,o:Lactobacillales,f:Lactobacillaceae,g:Lactobacillus,s:Lactobacillus mucosae
33	OTU_1942:k:Bacteria,p:Bacteroidota,c:Bacteroidia,o:Chitinophagales,f:Chitinophagaceae
34	OTU_1353:k:Bacteria,p:Patescibacteria,c:Saccharimonadia,o:Saccharimonadales
35	OTU_4288:k:Bacteria,p:Firmicutes,c:Clostridia,o:Lachnospirales,f:Lachnospiraceae,g:Blautia,s:uncultured bacterium
36	OTU_4371:k:Bacteria,p:Firmicutes,c:Clostridia,o:Lachnospirales,f:Lachnospiraceae,g:Dorea,s:uncultured bacterium
37	OTU_2850:k:Bacteria,p:Proteobacteria,c:Alphaproteobacteria,o:Rhodobacterales,f:Rhodobacteraceae,g:Defluviimonas
38	OTU_3660:k:Bacteria,p:Firmicutes,c:Bacilli,o:Erysipelotrichales,f:Erysipelotrichaceae,g:Allobaculum
39	OTU_3643:k:Bacteria
40	OTU_3017:k:Bacteria,p:Firmicutes
41	OTU_3263:k:Bacteria
42	OTU_3704:k:Bacteria,p:Firmicutes,c:Bacilli,o:Erysipelotrichales,f:Erysipelotrichaceae,g:Holdemanella,s:uncultured
43	OTU_2962:k:Bacteria,p:Firmicutes,c:Bacilli
44	OTU_4031:k:Bacteria
45	OTU_3761:k:Bacteria,p:Firmicutes,c:Clostridia
46	OTU_3386:k:Bacteria,p:Campilobacterota,c:Campylobacteria,o:Campylobacterales,f:Helicobacteraceae,g:Helicobacter,s:Helicobacter muridarum
47	OTU_2188:k:Bacteria,p:Acidobacteriota,c:Acidobacteriae,o:Bryobacterales,f:Bryobacteraceae,g:Bryobacter,s:uncultured
48	OTU_4513:k:Bacteria,p:Proteobacteria,c:Alphaproteobacteria,o:Sphingomonadales,f:Sphingomonadaceae,g:Sphingobium

Supplementary Table S10. Shared OTUs among lung, stomach and gut groups

No.	Shared OUT descriptions
1	OTU_23:k:Bacteria,p:Proteobacteria,c:Gammaproteobacteria,o:Pasteurellales,f:Pasteurellaceae,g:Rodentibacter,s:Pasteurellaceae bacterium
2	OTU_14:k:Bacteria,p:Bacteroidota,c:Bacteroidia,o:Bacteroidales,f:Porphyromonadaceae,g:Porphyromonas,s:Uncultured bacterium
3	OTU_80:k:Bacteria,p:Firmicutes,c:Negativicutes,o:Veillonellales-Selenomonadales,f:Veillonellaceae,g:Veillonella,sUncultured Veillonellaceae
4	OTU_26:k:Bacteria,p:Actinobacteriota,c:Actinobacteria,o:Actinomycetales,f:Actinomycetaceae,g:Actinomyces
5	OTU_20:k:Bacteria,p:Actinobacteriota,c:Actinobacteria,o:Micrococcales,f:Micrococcaceae,g:Rothia,s:Uncultured
6	OTU_78:k:Bacteria,p:Fusobacteriota,c:Fusobacteriia,o:Fusobacteriales,f:Fusobacteriaceae,g:Fusobacterium

7	OTU_35:k:Bacteria,p:Proteobacteria,c:Gammaproteobacteria,o:Burkholderiales,f:Alcaligenaceae
8	OTU_114:k:Bacteria,p:Firmicutes,c:Bacilli,o:Lactobacillales,f:Streptococcaceae,g:Streptococcus,s:Uncultured bacterium
9	OTU_1859:k:Bacteria,p:Proteobacteria,c:Gammaproteobacteria,o:Pasteurellales,f:Pasteurellaceae,g:Rodentibacter,s:Pasteurellaceae bacterium
10	OTU_100:k:Bacteria,p:Proteobacteria,c:Gammaproteobacteria,o:Pasteurellales,f:Pasteurellaceae,g:Rodentibacter,s:Pasteurellaceae bacterium
11	OTU_116:k:Bacteria,p:Proteobacteria,c:Alphaproteobacteria,o:Rhizobiales,f:Xanthobacteraceae,g:Bradyrhizobium
12	OTU_76:k:Bacteria,p:Firmicutes,c:Bacilli,o:Lactobacillales,f:Streptococcaceae,g:Streptococcus,s:Streptococcus azizii
13	OTU_65:k:Bacteria,p:Bacteroidota,c:Bacteroidia,o:Bacteroidales,f:Bacteroidaceae,g:Bacteroides
14	OTU_12:k:Bacteria,p:Proteobacteria,c:Gammaproteobacteria,o:Enterobacterales,f:Enterobacteriaceae,g:Escherichia-Shigella, s:E. coli
15	OTU_215:k:Bacteria,p:Firmicutes,c:Clostridia,o:Lachnospirales,f:Lachnospiraceae
16	OTU_206:k:Bacteria,p:Actinobacteriota,c:Actinobacteria,o:Corynebacteriales,f:Corynebacteriaceae,g:Corynebacterium,s:Corynebacterium pseudotuberculosis
17	OTU_106:k:Bacteria,p:Proteobacteria,c:Gammaproteobacteria,o:Burkholderiales,f:Burkholderiaceae,g:Ralstonia,s:Ralstonia mannitolilytica
18	OTU_289:k:Bacteria,p:Firmicutes,c:Bacilli,o:Lactobacillales
19	OTU_477:k:Bacteria,p:Actinobacteriota,c:Actinobacteria
20	OTU_10:k:Bacteria,p:Firmicutes,c:Bacilli,o:Lactobacillales,f:Lactobacillaceae,g:Lactobacillus,s:Lactobacillus murinus
21	OTU_237:k:Bacteria,p:Firmicutes,c:Clostridia,o:Peptostreptococcales-Tissierellales,f:Peptostreptococcaceae,g:Peptostreptococcus,s:Peptostreptococcus sp
22	OTU_754:k:Bacteria,p:Firmicutes,c:Bacilli,o:Erysipelotrichales,f:Erysipelotrichaceae,g:Dielma,s:uncultured bacterium
23	OTU_72:k:Bacteria,p:Firmicutes,c:Bacilli,o:Lactobacillales,f:Aerococcaceae
24	OTU_141:k:Bacteria,p:Actinobacteriota,c:Actinobacteria,o:Corynebacteriales,f:Corynebacteriaceae,g:Corynebacterium,s:Corynebacterium zhongnanshanii
25	OTU_44:k:Bacteria,p:Firmicutes,c:Bacilli,o:Staphylococcales,f:Staphylococcaceae,g:Staphylococcus,s:Staphylococcus fleurettii
26	OTU_2913:k:Bacteria,p:Proteobacteria,c:Gammaproteobacteria,o:Pasteurellales,f:Pasteurellaceae,g:Rodentibacter,s:Pasteurellaceae bacterium
27	OTU_3053:k:Bacteria,p:Proteobacteria,c:Gammaproteobacteria,o:Pseudomonadales,f:Moraxellaceae,g:Acinetobacter,s:Acinetobacter lwoffii
28	OTU_315:k:Bacteria,p:Firmicutes,c:Clostridia,o:Lachnospirales,f:Lachnospiraceae,g:[Eubacterium]-fissicatena group
29	OTU_39:k:Bacteria,p:Firmicutes,c:Bacilli,o:Staphylococcales,f:Gemellaceae,g:Gemella,s:uncultured bacterium
30	OTU_558:k:Bacteria,p:Proteobacteria,c:Gammaproteobacteria,o:Xanthomonadales,f:Xanthomonadaceae,g:Stenotrophomonas,s:Stenotrophomonas maltophilia
31	OTU_97:k:Bacteria,p:Firmicutes,c:Bacilli,o:Lactobacillales,f:Enterococcaceae,g:Enterococcus,s:Enterococcus faecium
32	OTU_241:k:Bacteria,p:Firmicutes,c:Bacilli,o:Lactobacillales
33	OTU_645:k:Bacteria,p:Actinobacteriota,c:Actinobacteria,o:Actinomycetales,f:Actinomycetaceae,g:Actinomyces,s:Uncultured
34	OTU_15:k:Bacteria,p:Firmicutes,c:Bacilli,o:Staphylococcales,f:Staphylococcaceae,g:Macrococcus,s:Macrococcus goetzii
35	OTU_124:k:Bacteria,p:Bacteroidota,c:Bacteroidia,o:Bacteroidales,f:Muribaculaceae
36	OTU_92:k:Bacteria,p:Firmicutes,c:Bacilli,o:Lactobacillales,f:Streptococcaceae,g:Lactococcus,s:Lactococcus cremoris

37 OTU_380:k:Bacteria,p:Fusobacteriota,c:Fusobacteriia,o:Fusobacteriales,f:Fusobacteriaceae,g:Fusobacterium
38 OTU_3:k:Bacteria,p:Proteobacteria,c:Gammaproteobacteria,o:Burkholderiales,f:Alcaligenaceae,g:Bordetella,s:Bordetella hinzii
39 OTU_516:k:Bacteria,p:Patescibacteria,c:Saccharimonadia,o:Saccharimonadales
40 OTU_91:k:Bacteria,p:Proteobacteria,c:Alphaproteobacteria,o:Sphingomonadales,f:Sphingomonadaceae,g:Sphingomonas,s:Sphingomonas kyeonggiensis
41 OTU_13:k:Bacteria,p:Firmicutes,c:Bacilli,o:Lactobacillales,f:Streptococcaceae,g:Streptococcus,s:Streptococcus macedonicus
42 OTU_614:k:Bacteria,p:Firmicutes,c:Bacilli,o:Mycoplasmatales,f:Mycoplasmataceae,g:Mycoplasma,s:uncultured
43 OTU_41:k:Bacteria,p:Proteobacteria,c:Gammaproteobacteria,o:Pseudomonadales,f:Moraxellaceae,g:Acinetobacter
44 OTU_56:k:Bacteria,p:Proteobacteria,c:Gammaproteobacteria,o:Pasteurellales,f:Pasteurellaceae
45 OTU_1209:k:Bacteria,p:Proteobacteria,c:Alphaproteobacteria,o:Rhizobiales,f:Rhizobiaceae,g:Bartonella,s:Uncultured
46 OTU_556:k:Bacteria,p:Firmicutes,c:Clostridia,o:Lachnospirales,f:Lachnospiraceae
47 OTU_133:k:Bacteria,p:Actinobacteriota,c:Actinobacteria,o:Micrococcales,f:Dermabacteraceae,g:Brachybacterium,s:Uncultured
48 OTU_1011:k:Bacteria,p:Firmicutes,c:Bacilli,o:Bacillales,f:Bacillaceae,g:Bacillus,s:Bacillus altitudinis
49 OTU_801:k:Bacteria,p:Firmicutes,c:Bacilli,o:Lactobacillales,f:Vagococcaceae,g:Vagococcus,s:Vagococcus fluvialis
50 OTU_258:k:Bacteria,p:Proteobacteria,c:Gammaproteobacteria,o:Pseudomonadales,f:Pseudomonadaceae,g:Pseudomonas,s:Pseudomonas mendocina
51 OTU_502:k:Bacteria,p:Bacteroidota,c:Bacteroidia,o:Bacteroidales,f:Bacteroidaceae,g:Bacteroides,s:Bacteroides thetaiotaomicron
52 OTU_1029:k:Bacteria,p:Firmicutes,c:Bacilli,o:Erysipelotrichales,f:Erysipelotrichaceae
53 OTU_112:k:Bacteria,p:Actinobacteriota,c:Actinobacteria,o:Micrococcales,f:Intrasporangiaceae,g:Janibacter,s:Janibacter melonis
54 OTU_805:k:Bacteria,p:Proteobacteria,c:Alphaproteobacteria,o:Rhodobacterales,f:Rhodobacteraceae,g:Gemmobacter
55 OTU_21:k:Bacteria,p:Fusobacteriota,c:Fusobacteriia,o:Fusobacteriales,f:Fusobacteriaceae,g:Fusobacterium
56 OTU_2324:k:Bacteria,p:Firmicutes,c:Bacilli,o:Bacillales,f:Bacillaceae,g:Bacillus,s:Peribacillus frigoritolerans
57 OTU_243:k:Bacteria,p:Proteobacteria,c:Gammaproteobacteria,o:Pseudomonadales,f:Moraxellaceae,g:Enhydrobacter
58 OTU_1:k:Bacteria,p:Firmicutes,c:Bacilli,o:Lactobacillales,f:Lactobacillaceae,g:Lactobacillus,s:Lactobacillus johnsonii
59 OTU_22:k:Bacteria,p:Bacteroidota,c:Bacteroidia,o:Bacteroidales,f:Prevotellaceae,g:Prevotella,s:Uncultured Prevotella
60 OTU_98:k:Bacteria,p:Firmicutes,c:Clostridia,o:Oscillospirales,f:Ruminococcaceae,g:Subdoligranulum
61 OTU_3520:k:Bacteria,p:Bacteroidota,c:Bacteroidia,o:Cytophagales,f:Microscillaceae,g:uncultured,s:uncultured bacterium
62 OTU_28:k:Bacteria,p:Firmicutes,c:Clostridia,o:Lachnospirales,f:Lachnospiraceae,g:Tyzzerella,s:uncultured bacterium
63 OTU_58:k:Bacteria,p:Firmicutes,c:Clostridia,o:Lachnospirales,f:Lachnospiraceae,g:[Ruminococcus]-gauvreauii group
64 OTU_54:k:Bacteria,p:Proteobacteria,c:Gammaproteobacteria,o:Enterobacterales,f:Enterobacteriaceae,g:Enterobacter,s:Enterobacter roggenkampii
65 OTU_1149:k:Bacteria,p:Firmicutes,c:Bacilli,o:Lactobacillales,f:Lactobacillaceae,g:Lactobacillus,s:Lactobacillus acidophilus
66 OTU_334:k:Bacteria,p:Firmicutes,c:Bacilli,o:Erysipelotrichales,f:Erysipelatoclostridiaceae,g:Coprobacillus,s:Coprobacillus cateniformis
67 OTU_373:k:Bacteria,p:Firmicutes,c:Clostridia,o:Oscillospirales,f:Oscillospiraceae,g:NK4A214 group

OTU_33:k:Bacteria,p:Firmicutes,c:Clostridia,o:Lachnospirales,f:Lachnospiraceae,g:[Ruminococcus]-gnavus group
OTU_4:k:Bacteria,p:Firmicutes,c:Bacilli,o:Lactobacillales,f:Lactobacillaceae,g:Lactobacillus,s:Limosilactobacillus reuteri
OTU_9:k:Bacteria,p:Campilobacterota,c:Campylobacteria,o:Campylobacterales,f:Helicobacteraceae,g:Helicobacter,s:Helicobacter apodemus
OTU_16:k:Bacteria,p:Actinobacteriota,c:Coriobacteriia,o:Coriobacteriales,f:Atopobiaceae,g:Olsenella,s:uncultured
OTU_7:k:Bacteria,p:Actinobacteriota,c:Actinobacteria,o:Bifidobacteriales,f:Bifidobacteriaceae,g:Bifidobacterium,s:Bifidobacterium faecale
OTU_60:k:Bacteria,p:Firmicutes,c:Bacilli,o:Erysipelotrichales,f:Erysipelotrichaceae,g:Allobaculum,s:uncultured bacterium
OTU_17:k:Bacteria,p:Firmicutes,c:Bacilli,o:Lactobacillales,f:Lactobacillaceae,g:Lactobacillus,s:Limosilactobacillus mucosae
OTU_40:k:Bacteria,p:Actinobacteriota,c:Actinobacteria,o:Bifidobacteriales,f:Bifidobacteriaceae,g:Bifidobacterium,s:Bifidobacterium longum
OTU_61:k:Bacteria,p:Firmicutes,c:Negativicutes,o:Veillonellales-Selenomonadales,f:Veillonellaceae,g:Megasphaera
OTU_24:k:Bacteria,p:Deferribacterota,c:Deferribacteres,o:Deferribacterales,f:Deferribacteraceae,g:Mucispirillum
OTU_47:k:Bacteria,p:Firmicutes,c:Bacilli,o:Lactobacillales,f:Lactobacillaceae,g:Lactobacillus,s:Lactobacillus ruminis
OTU_18:k:Bacteria,p:Actinobacteriota,c:Actinobacteria,o:Bifidobacteriales,f:Bifidobacteriaceae,g:Bifidobacterium,s:Bifidobacterium pseudolongum
OTU_506:k:Bacteria,p:Firmicutes,c:Negativicutes,o:Veillonellales-Selenomonadales,f:Veillonellaceae,g:Dialister,s:Dialister succinatiphilus
OTU_34:k:Bacteria,p:Firmicutes,c:Bacilli,o:Erysipelotrichales,f:Erysipelotrichaceae,g:Allobaculum,s:Uncultured bacterium
OTU_616:k:Bacteria,p:Firmicutes,c:Bacilli,o:Lactobacillales,f:Lactobacillaceae,g:Lactobacillus,s:Lactobacillus jensenii
OTU_31:k:Bacteria,p:Firmicutes,c:Clostridia,o:Peptostreptococcales-Tissierellales,f:Peptostreptococcaceae,g:Romboutsia
OTU_45:k:Bacteria,p:Proteobacteria,c:Gammaproteobacteria,o:Burkholderiales,f:Sutterellaceae,g:Parasutterella
OTU_135:k:Bacteria,p:Proteobacteria,c:Gammaproteobacteria,o:Aeromonadales,f:Succinivibrionaceae,g:Anaerobiospirillum
OTU_48:k:Bacteria,p:Spirochaetota,c:Spirochaetia,o:Spirochaetales,f:Spirochaetaceae,g:Treponema,s:Uncultured
OTU_8:k:Bacteria,p:Campilobacterota,c:Campylobacteria,o:Campylobacterales,f:Helicobacteraceae,g:Helicobacter,s:Helicobacter muridarum
OTU_46:k:Bacteria,p:Bacteroidota,c:Bacteroidia,o:Bacteroidales,f:Prevotellaceae,g:Alloprevotella,s:uncultured bacterium
OTU_6:k:Bacteria,p:Firmicutes,c:Bacilli,o:Erysipelotrichales,f:Erysipelotrichaceae,g:Turicibacter,s:Turicibacter LA61
OTU_2466:k:Bacteria,p:Firmicutes,c:Bacilli,o:Erysipelotrichales,f:Erysipelatoclostridiaceae,g:Catenibacterium
OTU_52:k:Bacteria,p:Actinobacteriota,c:Coriobacteriia,o:Coriobacteriales,f:Coriobacteriaceae,g:Collinsella,s:uncultured_bacterium
OTU_2617:k:Bacteria,p:Firmicutes,c:Clostridia,o:Lachnospirales,f:Lachnospiraceae
OTU_119:k:Bacteria,p:Actinobacteriota,c:Coriobacteriia,o:Coriobacteriales,f:Atopobiaceae
OTU_5:k:Bacteria,p:Firmicutes,c:Bacilli,o:Lactobacillales,f:Lactobacillaceae,g:Lactobacillus,s:Lactobacillus intestinalis
OTU_2552:k:Bacteria,p:Firmicutes,c:Clostridia,o:Lachnospirales,f:Lachnospiraceae
OTU_121:k:Bacteria,p:Firmicutes,c:Negativicutes,o:Acidaminococcales,f:Acidaminococcaceae,g:Phascolarctobacterium,s:uncultured
OTU_64:k:Bacteria,p:Firmicutes,c:Clostridia,o:Clostridiales,f:Clostridiaceae,g:Clostridium-sensu-stricto-1,s:Clostridium perfringens
OTU_73:k:Bacteria,p:Bacteroidota,c:Bacteroidia,o:Bacteroidales,f:Bacteroidaceae,g:Bacteroides

99 OTU_79:k:Bacteria,p:Actinobacteriota,c:Actinobacteria,o:Micrococcales,f:Micrococcaceae,g:Glutamicibacter
100 OTU_676:k:Bacteria,p:Campilobacterota,c:Campylobacteria,o:Campylobacterales,f:Helicobacteraceae,g:Helicobacter,s:Helicobacter japonicus
101 OTU_150:k:Bacteria,p:Firmicutes,c:Bacilli,o:Erysipelotrichales,f:Erysipelotrichaceae,g:uncultured,s:uncultured bacterium
102 OTU_623:k:Bacteria,p:Actinobacteriota,c:Actinobacteria,o:Micrococcales,f:Micrococcaceae,g:Kocuria,s:Kocuria palustris
103 OTU_77:k:Bacteria,p:Firmicutes,c:Bacilli,o:Erysipelotrichales,f:Erysipelotrichaceae,g:uncultured,s:uncultured bacterium
104 OTU_30:k:Bacteria,p:Firmicutes,c:Clostridia,o:Clostridiales,f:Clostridiaceae,g:Clostridium-sensu-stricto-1,s:Clostridium disporicum
105 OTU_313:k:Bacteria,p:Firmicutes,c:Bacilli,o:Erysipelotrichales,f:Erysipelotrichaceae,g:Turicibacter,s:Turicibacter sanguinis
106 OTU_283:k:Bacteria,p:Spirochaetota,c:Brachyspirae,o:Brachyspirales,f:Brachyspiraceae,g:Brachyspira
107 OTU_168:k:Bacteria,p:Desulfobacterota,c:Desulfovibrionia,o:Desulfovibrionales,f:Desulfovibrionaceae,g:uncultured
108 OTU_75:k:Bacteria,p:Bacteroidota,c:Bacteroidia,o:Bacteroidales,f:Muribaculaceae
109 OTU_2897:k:Bacteria,p:Bacteroidota,c:Bacteroidia,o:Bacteroidales,f:Prevotellaceae,g:Prevotella,s:uncultured
110 OTU_36:k:Bacteria,p:Firmicutes,c:Clostridia,o:Lachnospirales,f:Lachnospiraceae,g:Blautia,s:uncultured bacterium
111 OTU_37:k:Bacteria,p:Campilobacterota,c:Campylobacteria,o:Campylobacterales,f:Helicobacteraceae,g:Helicobacter,s:Helicobacter cholecystus
112 OTU_42:k:Bacteria,p:Firmicutes,c:Bacilli,o:Lactobacillales,f:Streptococcaceae,g:Streptococcus,s:Streptococcus hyointestinalis
113 OTU_104:k:Bacteria,p:Desulfobacterota,c:Desulfovibrionia,o:Desulfovibrionales,f:Desulfovibrionaceae,g:Desulfovibrio,s:uncultured
114 OTU_86:k:Bacteria,p:Firmicutes,c:Clostridia,o:Lachnospirales,f:Lachnospiraceae,g:Roseburia,s:Uncultured bacterium
115 OTU_695:k:Bacteria,p:Firmicutes,c:Clostridia,o:Peptostreptococcales-Tissierellales,f:Anaerovoracaceae,g:Family-XIII-AD3011 group,s:uncultured
116 OTU_222:k:Bacteria,p:Proteobacteria,c:Gammaproteobacteria,o:Aeromonadales,f:Succinivibrionaceae,g:Succinivibrio,s:uncultured
117 OTU_518:k:Bacteria,p:Firmicutes,c:Clostridia,o:Clostridiales,f:Clostridiaceae,g:Sarcina
118 OTU_2083:k:Bacteria,p:Firmicutes,c:Clostridia,o:Lachnospirales,f:Lachnospiraceae,g:Blautia,s:Uncultured bacterium
119 OTU_254:k:Bacteria,p:Actinobacteriota,c:Coriobacteriia,o:Coriobacteriales,f:Coriobacteriaceae,g:Collinsella,s:Collinsella BA40
120 OTU_29:k:Bacteria,p:Firmicutes,c:Clostridia,o:Lachnospirales,f:Lachnospiraceae,g:Blautia,s:Blautia pseudococcoides
121 OTU_772:k:Bacteria,p:Actinobacteriota,c:Actinobacteria,o:Corynebacteriales,f:Corynebacteriaceae,g:Corynebacterium,s:Uncultured Corynebacterium
122 OTU_126:k:Bacteria,p:Bacteroidota,c:Bacteroidia,o:Bacteroidales,f:Muribaculaceae
123 OTU_162:k:Bacteria,p:Firmicutes,c:Clostridia,o:Oscillospirales,f:Ruminococcaceae,g:Faecalibacterium,s:uncultured bacterium
124 OTU_196:k:Bacteria,p:Firmicutes,c:Negativicutes,o:Veillonellales-Selenomonadales,f:Veillonellaceae,g:Megasphaera
125 OTU_90:k:Bacteria,p:Bacteroidota,c:Bacteroidia,o:Bacteroidales,f:Bacteroidaceae,g:Bacteroides,s:uncultured bacterium
126 OTU_143:k:Bacteria,p:Firmicutes,c:Clostridia,o:Lachnospirales,f:Lachnospiraceae,g:Marvinbryantia,s:uncultured bacterium
127 OTU_50:k:Bacteria,p:Proteobacteria,c:Gammaproteobacteria,o:Pasteurellales,f:Pasteurellaceae,g:Actinobacillus
128 OTU_253:k:Bacteria,p:Bacteroidota,c:Bacteroidia,o:Bacteroidales,f:Tannerellaceae,g:Parabacteroides
129 OTU_365:k:Bacteria,p:Firmicutes,c:Negativicutes,o:Veillonellales-Selenomonadales,f:Selenomonadaceae,g:Mitsuokella

OTU_740:k:Bacteria,p:Firmicutes,c:Clostridia,o:Oscillospirales,f:Oscillospiraceae,g:uncultured
OTU_88:k:Bacteria,p:Firmicutes,c:Bacilli,o:Erysipelotrichales,f:Erysipelotrichaceae,g:Allobaculum
OTU_179:k:Bacteria,p:Proteobacteria,c:Gammaproteobacteria,o:Burkholderiales,f:Sutterellaceae,g:Sutterella
OTU_3478:k:Bacteria,p:Firmicutes,c:Clostridia,o:Lachnospirales,f:Lachnospiraceae,g:Blautia,s:uncultured bacterium
OTU_371:k:Bacteria,p:Firmicutes,c:Clostridia,o:Lachnospirales,f:Lachnospiraceae,g:uncultured
OTU_3461:k:Bacteria,p:Firmicutes,c:Clostridia,o:Oscillospirales,f:Oscillospiraceae,g:uncultured
OTU_343:k:Bacteria,p:Deferribacterota,c:Deferribacteres,o:Deferribacterales,f:Deferribacteraceae,g:Mucispirillum
OTU_3964:k:Bacteria,p:Actinobacteriota,c:Actinobacteria,o:Micrococcales,f:Micrococcaceae,g:Neomicrococcus,s:Micrococcus lylae
OTU_1644:k:Bacteria,p:Firmicutes,c:Clostridia,o:Lachnospirales,f:Lachnospiraceae
OTU_87:k:Bacteria,p:Proteobacteria,c:Alphaproteobacteria,o:Paracaedibacterales,f:Paracaedibacteraceae,g:uncultured,s:uncultured
OTU_53:k:Bacteria,p:Firmicutes,c:Clostridia,o:Lachnospirales,f:Lachnospiraceae,g:Dorea
OTU_113:k:Bacteria,p:Firmicutes,c:Clostridia,o:Lachnospirales,f:Lachnospiraceae,g:Frisingicoccus,s:Frisingicoccus caecimuris
OTU_453:k:Bacteria,p:Proteobacteria,c:Alphaproteobacteria,o:Rhodobacterales,f:Rhodobacteraceae,g:Paracoccus
OTU_194:k:Bacteria,p:Actinobacteriota,c:Coriobacteriia,o:Coriobacteriales,f:Atopobiaceae,g:Libanicoccus,s:uncultured bacterium
OTU_390:k:Bacteria,p:Bacteroidota,c:Bacteroidia,o:Bacteroidales,f:Muribaculaceae
OTU_137:k:Bacteria,p:Proteobacteria,c:Gammaproteobacteria,o:Pasteurellales,f:Pasteurellaceae,g:Actinobacillus,s:Actinobacillus porcitonsillarum
OTU_613:k:Bacteria,p:Actinobacteriota,c:Actinobacteria,o:Bifidobacteriales,f:Bifidobacteriaceae,g:Bifidobacterium,s:Bifidobacterium bifidum
OTU_57:k:Bacteria,p:Bacteroidota,c:Bacteroidia,o:Bacteroidales,f:Bacteroidaceae,g:Bacteroides,s:uncultured bacterium
OTU_202:k:Bacteria,p:Firmicutes,c:Clostridia,o:Oscillospirales,f:Oscillospiraceae,g:uncultured
OTU_261:k:Bacteria,p:Bacteroidota,c:Bacteroidia,o:Bacteroidales,f:Bacteroidaceae,g:Bacteroides
OTU_642:k:Bacteria,p:Firmicutes,c:Clostridia,o:Lachnospirales,f:Lachnospiraceae,g:Blautia,s:uncultured
OTU_2551:k:Bacteria,p:Firmicutes
OTU_122:k:Bacteria,p:Firmicutes,c:Clostridia,o:Oscillospirales,f:Butyricicoccaceae,g:Butyricicoccus
OTU_808:k:Bacteria,p:Firmicutes,c:Clostridia,o:Lachnospirales,f:Lachnospiraceae,g:Lachnoclostridium,s:Uncultured bacterium
OTU_482:k:Bacteria,p:Proteobacteria,c:Alphaproteobacteria,o:Rhizobiales,f:Rhizobiaceae,g:Allorhizobium-Neorhizobium-Pararhizobium Rhizobium
OTU_400:k:Bacteria,p:Bacteroidota,c:Bacteroidia,o:Bacteroidales,f:Muribaculaceae
OTU_74:k:Bacteria,p:Firmicutes,c:Clostridia,o:Peptostreptococcales-Tissierellales,f:Peptostreptococcaceae,g:Peptoclostridium,s:Peptacetobacter hiranonis
OTU_1147:k:Bacteria,p:Firmicutes,c:Clostridia,o:Oscillospirales,f:[Eubacterium]_coprostanoligenes-group
OTU_279:k:Bacteria,p:Firmicutes,c:Clostridia,o:Peptostreptococcales-Tissierellales,f:Anaerovoracaceae,g:[Eubacterium]-nodatum group
OTU_208:k:Bacteria,p:Firmicutes,c:Clostridia,o:Oscillospirales,f:Ruminococcaceae,g:Fournierella
OTU_107:k:Bacteria,p:Firmicutes,c:Clostridia,o:Lachnospirales,f:Lachnospiraceae,g:Blautia,s:uncultured_bacterium

161 OTU_263:k:Bacteria,p:Bacteroidota,c:Bacteroidia,o:Bacteroidales,f:Muribaculaceae
162 OTU_156:k:Bacteria,p:Actinobacteriota,c:Coriobacteriia,o:Coriobacteriales,f:Coriobacteriaceae,g:Collinsella,s:Collinsella intestinalis
163 OTU_668:k:Bacteria,p:Desulfobacterota,c:Desulfovibrionia,o:Desulfovibrionales,f:Desulfovibrionaceae,g:Desulfovibrio,s:Uncultured
164 OTU_765:k:Bacteria,p:Actinobacteriota,c:Coriobacteriia,o:Coriobacteriales,f:Atopobiaceae,g:Olsenella
165 OTU_4315:k:Bacteria,p:Firmicutes,c:Bacilli,o:Lactobacillales,f:Lactobacillaceae,g:Lactobacillus,s:Uncultured bacterium
166 OTU_3197:k:Bacteria,p:Firmicutes,c:Bacilli,o:Lactobacillales,f:Lactobacillaceae,g:Lactobacillus,s:Uncultured bacterium
167 OTU_70:k:Bacteria,p:Bacteroidota,c:Bacteroidia,o:Bacteroidales,f:Prevotellaceae,g:Prevotellaceae NK3B31 group,s:uncultured
168 OTU_4237:k:Bacteria,p:Firmicutes,c:Bacilli,o:Lactobacillales,f:Lactobacillaceae,g:Lactobacillus,s:uncultured
169 OTU_118:k:Bacteria,p:Firmicutes,c:Clostridia,o:Lachnospirales,f:Lachnospiraceae,g:Fusicatenibacter
170 OTU_569:k:Bacteria,p:Bacteroidota,c:Bacteroidia,o:Bacteroidales,f:Bacteroidaceae,g:Bacteroides
171 OTU_440:k:Bacteria,p:Firmicutes,c:Clostridia,o:Lachnospirales,f:Lachnospiraceae,g:Tyzzerella
172 OTU_227:k:Bacteria,p:Firmicutes,c:Clostridia,o:Oscillospirales,f:Ruminococcaceae,g:Anaerotruncus
173 OTU_84:k:Bacteria,p:Bacteroidota,c:Bacteroidia,o:Bacteroidales,f:Muribaculaceae
174 OTU_280:k:Bacteria,p:Bacteroidota,c:Bacteroidia,o:Bacteroidales,f:Bacteroidaceae,g:Bacteroides
175 OTU_758:k:Bacteria,p:Firmicutes,c:Clostridia,o:Oscillospirales,f:Ruminococcaceae,g:Paludicola,s:uncultured
176 OTU_672:k:Bacteria,p:Firmicutes,c:Clostridia,o:Lachnospirales,f:Lachnospiraceae
177 OTU_2598:k:Bacteria,p:Firmicutes,c:Bacilli,o:Lactobacillales,f:Lactobacillaceae,g:Lactobacillus,s:uncultured Clostridiales
178 OTU_499:k:Bacteria,p:Bacteroidota,c:Bacteroidia,o:Bacteroidales,f:Muribaculaceae
179 OTU_109:k:Bacteria,p:Firmicutes,c:Clostridia,o:Oscillospirales,f:Oscillospiraceae,g:UCG-005,s:uncultured bacterium
180 OTU_159:k:Bacteria,p:Firmicutes,c:Negativicutes,o:Veillonellales-Selenomonadales,f:Selenomonadaceae,g:Megamonas
181 OTU_321:k:Bacteria,p:Firmicutes,c:Clostridia,o:Lachnospirales,f:Lachnospiraceae,g:Lachnospira,s:uncultured organism
182 OTU_589:k:Bacteria,p:Firmicutes,c:Bacilli,o:Erysipelotrichales,f:Erysipelotrichaceae,g:Allobaculum
183 OTU_355:k:Bacteria,p:Firmicutes,c:Clostridia,o:Lachnospirales,f:Lachnospiraceae,g:Oribacterium,s:uncultured
184 OTU_110:k:Bacteria,p:Firmicutes,c:Clostridia,o:Lachnospirales,f:Lachnospiraceae,g:Roseburia,s:uncultured
185 OTU_4501:k:Bacteria,p:Firmicutes,c:Bacilli,o:Lactobacillales,f:Lactobacillaceae,g:Lactobacillus,s:Uncultured Lactobacillus
186 OTU_448:k:Bacteria,p:Firmicutes,c:Bacilli,o:Erysipelotrichales,f:Erysipelotrichaceae,g:Faecalibaculum,s:uncultured
187 OTU_312:k:Bacteria,p:Bacteroidota,c:Bacteroidia,o:Bacteroidales,f:Muribaculaceae
188 OTU_2565:k:Bacteria,p:Firmicutes,c:Clostridia,o:Lachnospirales,f:Lachnospiraceae,g:[Ruminococcus]-torques group,s:uncultured
189 OTU_3029:k:Bacteria
190 OTU_419:k:Bacteria,p:Bacteroidota,c:Bacteroidia,o:Bacteroidales,f:Bacteroidaceae,g:Bacteroides
191 OTU_766:k:Bacteria,p:Bacteroidota,c:Bacteroidia,o:Bacteroidales,f:Muribaculaceae

192 OTU_82:k:Bacteria,p:Firmicutes,c:Clostridia,o:Peptostreptococcales-Tissierellales,f:Peptostreptococcaceae

193 OTU_171:k:Bacteria,p:Firmicutes,c:Clostridia,o:Lachnospirales,f:Lachnospiraceae,g:Ruminococcus-torques group

194 OTU_342:k:Bacteria,p:Firmicutes,c:Bacilli,o:Erysipelotrichales,f:Erysipelotrichaceae,g:Holdemanella,s:uncultured

195 OTU_458:k:Bacteria,p:Bacteroidota,c:Bacteroidia,o:Bacteroidales,f:Marinifilaceae,g:Butyricimonas,s:Butyricimonas Marseille-P2440

196 OTU_3781:k:Bacteria,p:Firmicutes,c:Bacilli

197 OTU_166:k:Bacteria,p:Firmicutes,c:Clostridia,o:Lachnospirales,f:Lachnospiraceae,g:uncultured,s:uncultured bacterium

198 OTU_154:k:Bacteria,p:Bacteroidota,c:Bacteroidia,o:Bacteroidales,f:Rikenellaceae,g:Alistipes,s:uncultured bacterium

199 OTU_737:k:Bacteria,p:Firmicutes,c:Clostridia,o:Oscillospirales,f:Oscillospiraceae,g:UCG-002,s:uncultured bacterium

200 OTU_89:k:Bacteria,p:Firmicutes,c:Clostridia,o:Oscillospirales,f:Ruminococcaceae,g:Ruminococcus

201 OTU_3816:k:Bacteria,p:Firmicutes,c:Clostridia,o:Lachnospirales,f:Lachnospiraceae

202 OTU_169:k:Bacteria,p:Firmicutes,c:Clostridia,o:Oscillospirales,f:Ruminococcaceae,g:Negativibacillus

203 OTU_19:k:Bacteria,p:Firmicutes,c:Bacilli,o:Mycoplasmatales,f:Mycoplasmataceae,g:Mycoplasma,s:Metamycoplasma sualvi

204 OTU_49:k:Bacteria,p:Firmicutes,c:Bacilli,o:Lactobacillales,f:Leuconostocaceae,g:Weissella,s:Weissella hellenica

205 OTU_281:k:Bacteria,p:Firmicutes,c:Bacilli,o:Erysipelotrichales,f:Erysipelatoclostridiaceae,g:Catenibacterium,s:uncultured bacterium

206 OTU_181:k:Bacteria,p:Firmicutes,c:Bacilli,o:Lactobacillales,f:Carnobacteriaceae,g:Trichococcus

207 OTU_347:k:Bacteria,p:Actinobacteriota,c:Coriobacteriia,o:Coriobacteriales,f:Atopobiaceae,g:Olsenella

208 OTU_344:k:Bacteria,p:Bacteroidota,c:Bacteroidia,o:Bacteroidales,f:Muribaculaceae

209 OTU_607:k:Bacteria,p:Bacteroidota,c:Bacteroidia,o:Bacteroidales,f:Tannerellaceae,g:Parabacteroides

210 OTU_2876:k:Bacteria,p:Firmicutes,c:Clostridia,o:Clostridiales,f:Clostridiaceae,g:Clostridium sensu stricto-1,s:Clostridium paraputrificum

211 OTU_11:k:Bacteria,p:Proteobacteria,c:Gammaproteobacteria,o:Burkholderiales,f:Burkholderiaceae,g:Ralstonia,s:Ralstonia insidiosa

212 OTU_643:k:Bacteria,p:Firmicutes,c:Bacilli,o:Bacillales,f:Bacillaceae,g:Bacillus,s:Bacillus licheniformis

213 OTU_3241:k:Bacteria,p:Firmicutes,c:Bacilli,o:Lactobacillales,f:Lactobacillaceae,g:Lactobacillus,s:Uncultured bacterium

214 OTU_553:k:Bacteria,p:Proteobacteria,c:Alphaproteobacteria,o:Rhizobiales,f:Rhizobiaceae,g:Mesorhizobium

215 OTU_414:k:Bacteria,p:Firmicutes,c:Negativicutes,o:Veillonellales-Selenomonadales,f:Veillonellaceae,g:Dialister,s:uncultured bacterium

216 OTU_2970:k:Bacteria,p:Actinobacteriota,c:Coriobacteriia,o:Coriobacteriales,f:Coriobacteriaceae,g:Collinsella,s:uncultured

217 OTU_101:k:Bacteria,p:Firmicutes,c:Clostridia,o:Oscillospirales,f:[Eubacterium]-coprostanoligenes group

218 OTU_2822:k:Bacteria,p:Firmicutes,c:Clostridia,o:Lachnospirales,f:Lachnospiraceae

219 OTU_447:k:Bacteria,p:Firmicutes,c:Bacilli,o:Erysipelotrichales,f:Erysipelotrichaceae,g:Dubosiella,s:Dubosiella newyorkensis

220 OTU_147:k:Bacteria,p:Firmicutes,c:Bacilli,o:Lactobacillales,f:Streptococcaceae,g:Streptococcus,s:Streptococcus caballi

221 OTU_161:k:Bacteria,p:Bacteroidota,c:Bacteroidia,o:Bacteroidales,f:Porphyromonadaceae,g:Porphyromonas,s:uncultured

222 OTU_1965:k:Bacteria,p:Firmicutes,c:Clostridia,o:Oscillospirales,f:[Eubacterium]-coprostanoligenes group

OTU_120:k:Bacteria,p:Firmicutes,c:Clostridia,o:Lachnospirales,f:Lachnospiraceae,g:Sellimonas,s:uncultured bacterium
OTU_348:k:Bacteria,p:Firmicutes,c:Bacilli,o:Erysipelotrichales,f:Erysipelatoclostridiaceae,g:Erysipelatoclostridium,s:Thomasclavelia ramosa
OTU_273:k:Bacteria,p:Bacteroidota,c:Bacteroidia,o:Bacteroidales,f:Prevotellaceae,g:Prevotellaceae UCG-001,s:uncultured
OTU_4398:k:Bacteria,p:Firmicutes,c:Clostridia,o:Peptostreptococcales-Tissierellales,f:Peptostreptococcaceae,g:Intestinibacter,s:Clostridiaceae-DJF-VP56
OTU_62:k:Bacteria,p:Firmicutes,c:Bacilli,o:Staphylococcales,f:Staphylococcaceae,g:Staphylococcus,s:Staphylococcus equorum
OTU_3005:k:Bacteria,p:Firmicutes,c:Clostridia,o:Lachnospirales,f:Lachnospiraceae
OTU_27:k:Bacteria,p:Spirochaetota,c:Spirochaetia,o:Spirochaetales,f:Spirochaetaceae,g:Treponema,s:uncultured bacterium
OTU_269:k:Bacteria,p:Actinobacteriota,c:Coriobacteriia,o:Coriobacteriales,f:Eggerthellaceae,g:Senegalimassilia,s:Uncultured
OTU_1269:k:Bacteria,p:Firmicutes,c:Clostridia,o:Oscillospirales,f:Ruminococcaceae,g:uncultured,s:uncultured bacterium
OTU_148:k:Bacteria,p:Actinobacteriota,c:Actinobacteria,o:Micrococcales,f:Micrococcaceae,g:Rothia,s:Uncultured
OTU_299:k:Bacteria,p:Firmicutes,c:Clostridia,o:Lachnospirales,f:Lachnospiraceae,g:Epulopiscium
OTU_205:k:Bacteria,p:Firmicutes,c:Clostridia,o:Lachnospirales,f:Lachnospiraceae,g:Dorea
OTU_433:k:Bacteria,p:Firmicutes,c:Clostridia,o:Oscillospirales,f:Butyricicoccaceae,g:Butyricicoccus,s:uncultured bacterium
OTU_357:k:Bacteria,p:Firmicutes,c:Clostridia,o:Oscillospirales,f:Oscillospiraceae,g:Oscillospira,s:uncultured bacterium
OTU_800:k:Bacteria,p:Firmicutes,c:Clostridia,o:Peptostreptococcales-Tissierellales,f:Anaerovoracaceae,g:Family-XIII-AD3011
OTU_173:k:Bacteria,p:Bacteroidota,c:Bacteroidia,o:Bacteroidales,f:Rikenellaceae,g:Alistipes,s:uncultured Bacteroidales
OTU_1783:k:Bacteria,p:Bacteroidota,c:Bacteroidia,o:Bacteroidales,f:Bacteroidaceae,g:Bacteroides
OTU_235:k:Bacteria,p:Bacteroidota,c:Bacteroidia,o:Bacteroidales,f:Tannerellaceae,g:Parabacteroides,s:Parabacteroides sp.
OTU_298:k:Bacteria,p:Firmicutes,c:Clostridia,o:Oscillospirales,f:Oscillospiraceae,g:NK4A214 group,s:uncultured
OTU_117:k:Bacteria,p:Bacteroidota,c:Bacteroidia,o:Bacteroidales,f:Prevotellaceae,g:Alloprevotella,s:uncultured
OTU_823:k:Bacteria,p:Firmicutes,c:Clostridia,o:Lachnospirales,f:Lachnospiraceae
OTU_473:k:Bacteria,p:Firmicutes,c:Clostridia,o:Oscillospirales,f:Oscillospiraceae,g:UCG-005
OTU_549:k:Bacteria,p:Bacteroidota,c:Bacteroidia,o:Bacteroidales,f:Muribaculaceae
OTU_183:k:Bacteria,p:Actinobacteriota,c:Actinobacteria,o:Micrococcales,f:Dermacoccaceae,g:Dermacoccus
OTU_1175:k:Bacteria,p:Firmicutes,c:Clostridia,o:Oscillospirales,f:Oscillospiraceae,g:UCG-005,s:uncultured bacterium
OTU_3984:k:Bacteria,p:Firmicutes,c:Bacilli,o:Lactobacillales,f:Lactobacillaceae,g:Lactobacillus,s:Uncultured bacterium
OTU_322:k:Bacteria,p:Firmicutes,c:Clostridia,o:Lachnospirales,f:Lachnospiraceae,g:Anaerostipes
OTU_38:k:Bacteria,p:Proteobacteria,c:Gammaproteobacteria,o:Pseudomonadales,f:Pseudomonadaceae,g:Pseudomonas,s:Pseudomonas putida
OTU_304:k:Bacteria,p:Firmicutes,c:Bacilli,o:Erysipelotrichales,f:Erysipelotrichaceae,g:Solobacterium,s:uncultured
OTU_3486:k:Bacteria,p:Firmicutes,c:Clostridia,o:Lachnospirales,f:Lachnospiraceae,g:Blautia,s:uncultured
OTU_2511:k:Bacteria,p:Actinobacteriota,c:Actinobacteria,o:Corynebacteriales,f:Nocardiaceae,g:Gordonia,s:Gordonia malaquae

OTU_3970:k:Bacteria

OTU_1354:k:Bacteria,p:Firmicutes,c:Bacilli

OTU_520:k:Bacteria,p:Firmicutes,c:Clostridia,o:Peptostreptococcales-Tissierellales,f:Peptostreptococcaceae,g:Terrisporobacter,s:Terrisporobacter muris

OTU_287:k:Bacteria,p:Actinobacteriota,c:Actinobacteria,o:Corynebacteriales,f:Corynebacteriaceae,g:Corynebacterium,s:Uncultured

OTU_915:k:Bacteria,p:Firmicutes,c:Clostridia,o:Eubacteriales,f:Eubacteriaceae,g:Eubacterium,s:Eubacterium limosum

OTU_325:k:Bacteria,p:Actinobacteriota,c:Coriobacteriia,o:Coriobacteriales,f:Eggerthellaceae

OTU_2355:k:Bacteria,p:Actinobacteriota,c:Actinobacteria,o:Corynebacteriales,f:Corynebacteriaceae,g:Corynebacterium,s:Uncultured

OTU_59:k:Bacteria,p:Firmicutes,c:Bacilli,o:Bacillales,f:Planococcaceae,g:Kurthia,s:Kurthia populi

OTU_255:k:Bacteria,p:Firmicutes,c:Clostridia,o:Monoglobales,f:Monoglobaceae,g:Monoglobus,s:uncultured

OTU_358:k:Bacteria,p:Bacteroidota,c:Bacteroidia,o:Bacteroidales,f:Muribaculaceae

OTU_177:k:Bacteria,p:Firmicutes,c:Clostridia,o:Oscillospirales,f:Oscillospiraceae,g:Oscillibacter,s:uncultured bacterium

OTU_1078:k:Bacteria,p:Proteobacteria,c:Gammaproteobacteria,o:Burkholderiales,f:Rhodocyclaceae,g:Dechloromonas

OTU_604:k:Bacteria,p:Firmicutes,c:Clostridia,o:Christensenellales,f:Christensenellaceae,g:Christensenellaceae R-7 group,s:uncultured

OTU_718:k:Bacteria,p:Actinobacteriota,c:Actinobacteria,o:Corynebacteriales,f:Dietziaceae,g:Dietzia,s:Dietzia timorensis

OTU_238:k:Bacteria,p:Firmicutes,c:Bacilli,o:Staphylococcales,f:Staphylococcaceae,g:Jeotgalicoccus

OTU_682:k:Bacteria,p:Firmicutes,c:Clostridia,o:Lachnospirales,f:Lachnospiraceae,g:[Eubacterium]-hallii group

OTU_535:k:Bacteria,p:Bacteroidota,c:Bacteroidia,o:Bacteroidales,f:Muribaculaceae

OTU_455:k:Bacteria,p:Bacteroidota,c:Bacteroidia,o:Bacteroidales,f:Muribaculaceae

OTU_689:k:Bacteria,p:Bacteroidota,c:Bacteroidia,o:Bacteroidales,f:Tannerellaceae,g:Parabacteroides,s:uncultured bacterium

OTU_612:k:Bacteria,p:Firmicutes,c:Clostridia,o:Peptostreptococcales-Tissierellales,f:Anaerovoracaceae,g:Mogibacterium,s:uncultured

OTU_174:k:Bacteria,p:Firmicutes,c:Clostridia,o:Oscillospirales,f:Ruminococcaceae

OTU_178:k:Bacteria,p:Firmicutes,c:Clostridia,o:Oscillospirales,f:Oscillospiraceae,g:Colidextribacter

OTU_1929:k:Bacteria,p:Firmicutes,c:Clostridia,o:Peptococcales,f:Peptococcaceae,g:uncultured,s:uncultured bacterium

OTU_742:k:Bacteria,p:Bacteroidota,c:Bacteroidia,o:Bacteroidales,f:Prevotellaceae

OTU_594:k:Bacteria,p:Proteobacteria,c:Gammaproteobacteria,o:Pseudomonadales,f:Moraxellaceae

OTU_1819:k:Bacteria,p:Firmicutes,c:Clostridia,o:Lachnospirales,f:Lachnospiraceae,g:GCA-900066575,s:uncultured bacterium

OTU_1822:k:Bacteria,p:Firmicutes,c:Clostridia,o:Lachnospirales,f:Lachnospiraceae,g:Coprococcus,s:uncultured bacterium

OTU_225:k:Bacteria,p:Bacteroidota,c:Bacteroidia,o:Bacteroidales,f:Bacteroidaceae,g:Bacteroides,s:uncultured bacterium

OTU_424:k:Bacteria,p:Firmicutes,c:Clostridia,o:Oscillospirales,f:Ruminococcaceae,g:uncultured,s:uncultured bacterium

OTU_158:k:Bacteria,p:Bacteroidota,c:Bacteroidia,o:Bacteroidales,f:Prevotellaceae,g:Alloprevotella,s:uncultured bacterium

OTU_501:k:Bacteria,p:Firmicutes,c:Clostridia,o:Oscillospirales,f:[Eubacterium]_coprostanoligenes group

OTU_285:k:Bacteria,p:Bacteroidota,c:Bacteroidia,o:Bacteroidales,f:Rikenellaceae,g:Rikenellaceae RC9 gut group,s:uncultured

OTU_1892:k:Bacteria,p:Firmicutes,c:Clostridia,o:Lachnospirales,f:Lachnospiraceae,g:Coprococcus

OTU_93:k:Bacteria,p:Firmicutes,c:Clostridia,o:Clostridia_UCG-014

OTU_2542:k:Bacteria,p:Firmicutes,c:Clostridia,o:Lachnospirales,f:Lachnospiraceae,g:Oribacterium,s:uncultured bacterium

OTU_231:k:Bacteria,p:Patescibacteria,c:Saccharimonadia,o:Saccharimonadales,f:Saccharimonadaceae,g:Candidatus Saccharimonas,s:uncultured

OTU_423:k:Bacteria,p:Bacteroidota,c:Bacteroidia,o:Bacteroidales,f:Muribaculaceae

OTU_1447:k:Bacteria,p:Firmicutes,c:Clostridia,o:Lachnospirales,f:Lachnospiraceae,g:uncultured

OTU_3745:k:Bacteria,p:Firmicutes,c:Clostridia,o:Lachnospirales,f:Lachnospiraceae,g:Lachnospiraceae FCS020 group,s:uncultured

OTU_204:k:Bacteria,p:Bacteroidota,c:Bacteroidia,o:Bacteroidales,f:Muribaculaceae

OTU_878:k:Bacteria,p:Fusobacteriota,c:Fusobacteriia,o:Fusobacteriales,f:Leptotrichiaceae,g:Leptotrichia,s:Leptotrichia PG10

OTU_387:k:Bacteria,p:Bacteroidota,c:Bacteroidia,o:Bacteroidales,f:Prevotellaceae,g:Prevotellaceae UCG-003

OTU_284:k:Bacteria,p:Firmicutes,c:Clostridia,o:Oscillospirales,f:Ruminococcaceae,g:uncultured,s:uncultured bacterium

OTU_826:k:Bacteria,p:Firmicutes,c:Clostridia,o:Oscillospirales,f:[Eubacterium]_coprostanoligenes group

OTU_125:k:Bacteria,p:Firmicutes,c:Bacilli,o:Staphylococcales,f:Staphylococcaceae,g:Jeotgalicoccus,s:Jeotgalicoccus pinnipedialis

OTU_3646:k:Bacteria,p:Firmicutes,c:Bacilli,o:RF39

OTU_1823:k:Bacteria,p:Proteobacteria,c:Alphaproteobacteria,o:Rhizobiales,f:Rhizobiaceae

OTU_1411:k:Bacteria,p:Actinobacteriota,c:Coriobacteriia,o:Coriobacteriales,f:Eggerthellaceae,g:uncultured,s:uncultured bacterium

OTU_1018:k:Bacteria,p:Firmicutes,c:Negativicutes,o:Veillonellales-Selenomonadales,f:Veillonellaceae,g:Allisonella,s:Allisonella histaminiformans

OTU_744:k:Bacteria,p:Bacteroidota,c:Bacteroidia,o:Bacteroidales,f:Muribaculaceae

OTU_1082:k:Bacteria,p:Actinobacteriota,c:Coriobacteriia,o:Coriobacteriales

OTU_3059:k:Bacteria,p:Firmicutes,c:Clostridia,o:Lachnospirales,f:Lachnospiraceae

OTU_937:k:Bacteria,p:Firmicutes,c:Bacilli,o:Lactobacillales,f:Streptococcaceae,g:Streptococcus,s:Strep Ococcus ferus

OTU_1439:k:Bacteria,p:Actinobacteriota,c:Actinobacteria,o:Micrococcales,f:Microbacteriaceae,g:Leucobacter,s:Leucobacter sp.

OTU_908:k:Bacteria,p:Firmicutes,c:Clostridia,o:Oscillospirales,f:Oscillospiraceae,g:uncultured

OTU_309:k:Bacteria,p:Firmicutes,c:Clostridia,o:Oscillospirales,f:Ruminococcaceae,g:uncultured,s:uncultured bacterium

OTU_2544:k:Bacteria,p:Firmicutes,c:Clostridia,o:Oscillospirales,f:Oscillospiraceae,g:uncultured,s:uncultured bacterium

OTU_2833:k:Bacteria,p:Desulfobacterota,c:Desulfovibrionia,o:Desulfovibrionales,f:Desulfovibrionaceae,g:uncultured,s:uncultured

OTU_904:k:Bacteria,p:Firmicutes,c:Bacilli,o:RF39

OTU_1875:k:Bacteria,p:Firmicutes,c:Clostridia,o:Clostridiales,f:Clostridiaceae,g:Clostridium sensu stricto 13,s:Clostridium subterminale

OTU_401:k:Bacteria,p:Proteobacteria,c:Alphaproteobacteria,o:Caulobacterales,f:Caulobacteraceae,g:Brevundimonas

316 OTU_4253:k:Bacteria,p:Firmicutes,c:Clostridia,o:Lachnospirales,f:Lachnospiraceae
317 OTU_898:k:Bacteria,p:Firmicutes,c:Clostridia,o:Christensenellales,f:Christensenellaceae,g:Christensenellaceae R-7 group,s:uncultured
318 OTU_274:k:Bacteria,p:Firmicutes,c:Clostridia,o:Oscillospirales,f:Ruminococcaceae,g:Ruminococcus,s:uncultured
319 OTU_1213:k:Bacteria,p:Firmicutes,c:Clostridia,o:Oscillospirales,f:Ruminococcaceae,g:uncultured
320 OTU_1552:k:Bacteria,p:Firmicutes,c:Clostridia,o:Oscillospirales,f:Oscillospiraceae,g:Colidextribacter,s:Uncultured bacterium
321 OTU_393:k:Bacteria,p:Actinobacteriota,c:Coriobacteriia,o:Coriobacteriales,f:Eggerthellaceae,g:uncultured,s:uncultured
322 OTU_615:k:Bacteria,p:Firmicutes,c:Clostridia,o:Monoglobales,f:Monoglobaceae,g:Monoglobus,s:uncultured bacterium
323 OTU_1407:k:Bacteria,p:Firmicutes,c:Bacilli,o:Erysipelotrichales,f:Erysipelatoclostridiaceae,g:Erysipelatoclostridium,s:Uncultured bacterium
324 OTU_517:k:Bacteria,p:Firmicutes,c:Clostridia,o:Lachnospirales,f:Lachnospiraceae
325 OTU_2626:k:Bacteria,p:Firmicutes,c:Negativicutes,o:Veillonellales-Selenomonadales,f:Selenomonadaceae,g:Megamonas,s:Megamonas funiformis
326 OTU_1003:k:Bacteria,p:Firmicutes,c:Clostridia,o:Oscillospirales,f:Oscillospiraceae,g:UCG-005,s:uncultured bacterium
327 OTU_2976:k:Bacteria,p:Firmicutes,c:Bacilli,o:Lactobacillales,f:Streptococcaceae,g:Streptococcus,s:Streptococcus anginosus
328 OTU_331:k:Bacteria,p:Bacteroidota,c:Bacteroidia,o:Bacteroidales,f:Rikenellaceae,g:dgA-11 gut group,s:uncultured bacterium
329 OTU_786:k:Bacteria,p:Firmicutes,c:Clostridia,o:Peptostreptococcales-Tissierellales,f:Anaerovoracaceae,g:[Eubacterium]-brachy group,s:uncultured
330 OTU_3909:k:Bacteria,p:Firmicutes,c:Clostridia,o:Lachnospirales,f:Lachnospiraceae
331 OTU_688:k:Bacteria,p:Firmicutes,c:Bacilli,o:Erysipelotrichales,f:Erysipelotrichaceae,g:uncultured,s:uncultured bacterium
332 OTU_631:k:Bacteria,p:Firmicutes,c:Clostridia,o:Oscillospirales,f:Oscillospiraceae
333 OTU_481:k:Bacteria,p:Firmicutes,c:Clostridia,o:Lachnospirales,f:Lachnospiraceae,g:uncultured,s:uncultured bacterium
334 OTU_249:k:Bacteria,p:Firmicutes,c:Bacilli,o:Lactobacillales,f:Aerococcaceae,g:Aerococcus,s:Aerococcus viridans
335 OTU_1137:k:Bacteria,p:Firmicutes,c:Clostridia,o:uncultured
336 OTU_1833:k:Bacteria,p:Firmicutes,c:Clostridia,o:Oscillospirales,f:Oscillospiraceae,g:uncultured
337 OTU_630:k:Bacteria,p:Firmicutes,c:Clostridia,o:Oscillospirales,f:Ruminococcaceae,g:Incertae Sedis,s:Uncultured bacterium
338 OTU_1104:k:Bacteria,p:Firmicutes,c:Bacilli,o:Bacillales,f:Planococcaceae
339 OTU_3986:k:Bacteria,p:Firmicutes,c:Clostridia,o:Oscillospirales,f:[Eubacterium]-coprostanoligenes group
340 OTU_2968:k:Bacteria,p:Firmicutes,c:Clostridia,o:Lachnospirales,f:Lachnospiraceae
341 OTU_368:k:Bacteria,p:Firmicutes,c:Bacilli,o:Lactobacillales,f:Aerococcaceae
342 OTU_3830:k:Bacteria,p:Firmicutes,c:Clostridia,o:Lachnospirales,f:Lachnospiraceae
343 OTU_449:k:Bacteria,p:Firmicutes,c:Clostridia,o:Lachnospirales,f:Lachnospiraceae
344 OTU_509:k:Bacteria,p:Firmicutes,c:Clostridia,o:Christensenellales,f:Christensenellaceae,g:Christensenellaceae R-7 group
345 OTU_2606:k:Bacteria,p:Actinobacteriota,c:Actinobacteria,o:Corynebacteriales,f:Corynebacteriaceae,g:Corynebacterium,s:Corynebacterium casei
346 OTU_746:k:Bacteria,p:Firmicutes,c:Negativicutes,o:Veillonellales-Selenomonadales,f:Veillonellaceae,g:Veillonella,s:Veillonella sp

OTU_418:k:Bacteria,p:Firmicutes,c:Bacilli,o:Erysipelotrichales,f:Erysipelatoclostridiaceae,g:Erysipelatoclostridium,s:uncultured
OTU_426:k:Bacteria,p:Bacteroidota,c:Bacteroidia,o:Bacteroidales,f:Rikenellaceae,g:Rikenellaceae RC9 gut group,s:uncultured
OTU_500:k:Bacteria,p:Bacteroidota,c:Bacteroidia,o:Bacteroidales,f:Muribaculaceae
OTU_483:k:Bacteria,p:Proteobacteria,c:Gammaproteobacteria,o:Burkholderiales,f:Comamonadaceae,g:Delftia,s:Delftia acidovorans
OTU_384:k:Bacteria,p:Firmicutes,c:Clostridia,o:Oscillospirales
OTU_3775:k:Bacteria,p:Firmicutes,c:Bacilli,o:Lactobacillales,f:Lactobacillaceae,g:Lactobacillus,s:Lactobacillus reuteri
OTU_534:k:Bacteria,p:Firmicutes,c:Bacilli,o:Lactobacillales,f:Aerococcaceae,g:Aerosphaera,s:Fundicoccus ignavus
OTU_1028:k:Bacteria,p:Firmicutes,c:Clostridia,o:Peptococcales,f:Peptococcaceae,g:uncultured,s:uncultured bacterium
OTU_391:k:Bacteria,p:Firmicutes,c:Clostridia,o:Oscillospirales,f:Oscillospiraceae,g:Flavonifractor,s:Flavonifractor plautii
OTU_565:k:Bacteria,p:Firmicutes,c:Clostridia,o:Oscillospirales,f:Ruminococcaceae,g:UBA1819,s:Ruthenibacterium lactatiformans
OTU_385:k:Bacteria,p:Firmicutes,c:Clostridia,o:Peptostreptococcales-Tissierellales
OTU_649:k:Bacteria,p:Firmicutes,c:Clostridia,o:Christensenellales,f:Christensenellaceae,g:Christensenellaceae R-7 group,s:uncultured
OTU_3395:k:Bacteria,p:Firmicutes,c:Clostridia,o:Lachnospirales,f:Lachnospiraceae
OTU_1768:k:Bacteria,p:Actinobacteriota,c:Coriobacteriia,o:Coriobacteriales,f:Atopobiaceae
OTU_1123:k:Bacteria,p:Firmicutes,c:Bacilli,o:Erysipelotrichales,f:Erysipelotrichaceae,g:uncultured,s:uncultured bacterium
OTU_1042:k:Bacteria,p:Firmicutes,c:Bacilli,o:Erysipelotrichales,f:Erysipelotrichaceae,g:Erysipelotrichaceae UCG-006,s:uncultured
OTU_4419:k:Bacteria,p:Firmicutes,c:Bacilli,o:Lactobacillales,f:Lactobacillaceae,g:Lactobacillus,s:Uncultured bacterium
OTU_314:k:Bacteria,p:Firmicutes,c:Clostridia,o:Lachnospirales,f:Lachnospiraceae
OTU_882:k:Bacteria,p:Actinobacteriota,c:Coriobacteriia,o:Coriobacteriales,f:Eggerthellaceae,g:Enterorhabdus,s:uncultured
OTU_705:k:Bacteria,p:Actinobacteriota,c:Coriobacteriia,o:Coriobacteriales,f:Coriobacteriaceae,g:Collinsella
OTU_2147:k:Bacteria,p:Firmicutes,c:Clostridia,o:Oscillospirales,f:Ruminococcaceae,g:[Eubacterium]-siraeum group
OTU_1203:k:Bacteria,p:Actinobacteriota,c:Coriobacteriia,o:Coriobacteriales,f:Eggerthellaceae
OTU_640:k:Bacteria,p:Bacteroidota,c:Bacteroidia,o:Bacteroidales,f:Rikenellaceae,g:Alistipes,s:uncultured
OTU_822:k:Bacteria,p:Bacteroidota,c:Bacteroidia,o:Bacteroidales,f:Muribaculaceae
OTU_337:k:Bacteria,p:Firmicutes,c:Bacilli,o:Lactobacillales,f:Aerococcaceae,g:Facklamia,s:Uncultured
OTU_3310:k:Bacteria,p:Actinobacteriota,c:Actinobacteria,o:Frankiales,f:Sporichthyaceae,g:hgcI clade,s:uncultured bacterium
OTU_2237:k:Bacteria,p:Actinobacteriota,c:Coriobacteriia,o:Coriobacteriales,f:Eggerthellaceae
OTU_1063:k:Bacteria,p:Firmicutes,c:Clostridia,o:Christensenellales,f:Christensenellaceae,g:Christensenellaceae R-7 group,s:uncultured
OTU_537:k:Bacteria,p:Firmicutes,c:Bacilli,o:Lactobacillales,f:Carnobacteriaceae
OTU_709:k:Bacteria,p:Proteobacteria,c:Gammaproteobacteria,o:Enterobacterales,f:Yersiniaceae,g:Serratia,s:Serratia proteamaculans
OTU_3243:k:Bacteria,p:Firmicutes,c:Bacilli,o:Lactobacillales,f:Lactobacillaceae,g:Lactobacillus,s:uncultured bacterium

378	OTU_1346:k:Bacteria,p:Actinobacteriota,c:Actinobacteria,o:Actinomycetales,f:Actinomycetaceae,g:Actinomyces
379	OTU_4014:k:Bacteria,p:Proteobacteria,c:Alphaproteobacteria,o:Sphingomonadales,f:Sphingomonadaceae,g:Novosphingobium
380	OTU_404:k:Bacteria,p:Firmicutes,c:Bacilli,o:Erysipelotrichales,f:Erysipelatoclostridiaceae,g:Candidatus Stoquefichus
381	OTU_663:k:Bacteria,p:Firmicutes,c:Clostridia,o:Clostridia vadinBB60 group
382	OTU_941:k:Bacteria,p:Firmicutes,c:Clostridia,o:Oscillospirales,f:Oscillospiraceae,g:Oscillibacter,s:uncultured bacterium
383	OTU_563:k:Bacteria,p:Firmicutes,c:Clostridia,o:Lachnospirales,f:Lachnospiraceae,g:Lachnospiraceae NK4A136 group
384	OTU_1827:k:Bacteria,p:Firmicutes,c:Clostridia,o:Peptostreptococcales-Tissierellales,g:Anaerococcus,s:Anaerococcus nagyae
385	OTU_767:k:Bacteria,p:Proteobacteria,c:Alphaproteobacteria,o:Sphingomonadales,f:Sphingomonadaceae,g:Erythrobacter,s:Erythrobacter DHXJ15
386	OTU_812:k:Bacteria,p:Firmicutes,c:Clostridia,o:Oscillospirales,f:Butyricicoccaceae,g:UCG-009,s:uncultured bacterium
387	OTU_713:k:Bacteria,p:Firmicutes,c:Bacilli,o:RF39
388	OTU_1333:k:Bacteria,p:Firmicutes,c:Clostridia,o:Oscillospirales,f:Ruminococcaceae,g:uncultured,s:uncultured bacterium
389	OTU_3562:k:Bacteria,p:Patescibacteria,c:Saccharimonadia,o:Saccharimonadales
390	OTU_944:k:Bacteria,p:Proteobacteria,c:Alphaproteobacteria,o:Rhizobiales,f:Hyphomicrobiaceae,g:Pedomicrobium,s:Uncultured
391	OTU_1622:k:Bacteria,p:Patescibacteria,c:Saccharimonadia,o:Saccharimonadales
392	OTU_1506:k:Bacteria,p:Patescibacteria,c:Saccharimonadia,o:Saccharimonadales
393	OTU_874:k:Bacteria,p:Actinobacteriota,c:Actinobacteria,o:Actinomycetales,f:Actinomycetaceae,g:Actinomyces,s:Uncultured
394	OTU_1316:k:Bacteria,p:Actinobacteriota,c:Coriobacteriia,o:Coriobacteriales,f:Eggerthellaceae,g:Slackia,s:Slackia piriformis
395	OTU_4301:k:Bacteria,p:Firmicutes,c:Bacilli,o:Lactobacillales,f:Streptococcaceae
396	OTU_1460:k:Bacteria,p:Actinobacteriota,c:Actinobacteria,o:Bifidobacteriales,f:Bifidobacteriaceae,g:Bifidobacterium,s:Bifidobacterium mongoliense
