# Supplementary figures and images for: A Comparative Analysis of the Stomach, Gut, and Lung Microbiomes in Rattus norvegicus
Source: Microorganisms. 2023 Sep 21;11(9):2359. doi: 10.3390/microorganisms11092359 (PMC10534326; doi:10.3390/microorganisms11092359)

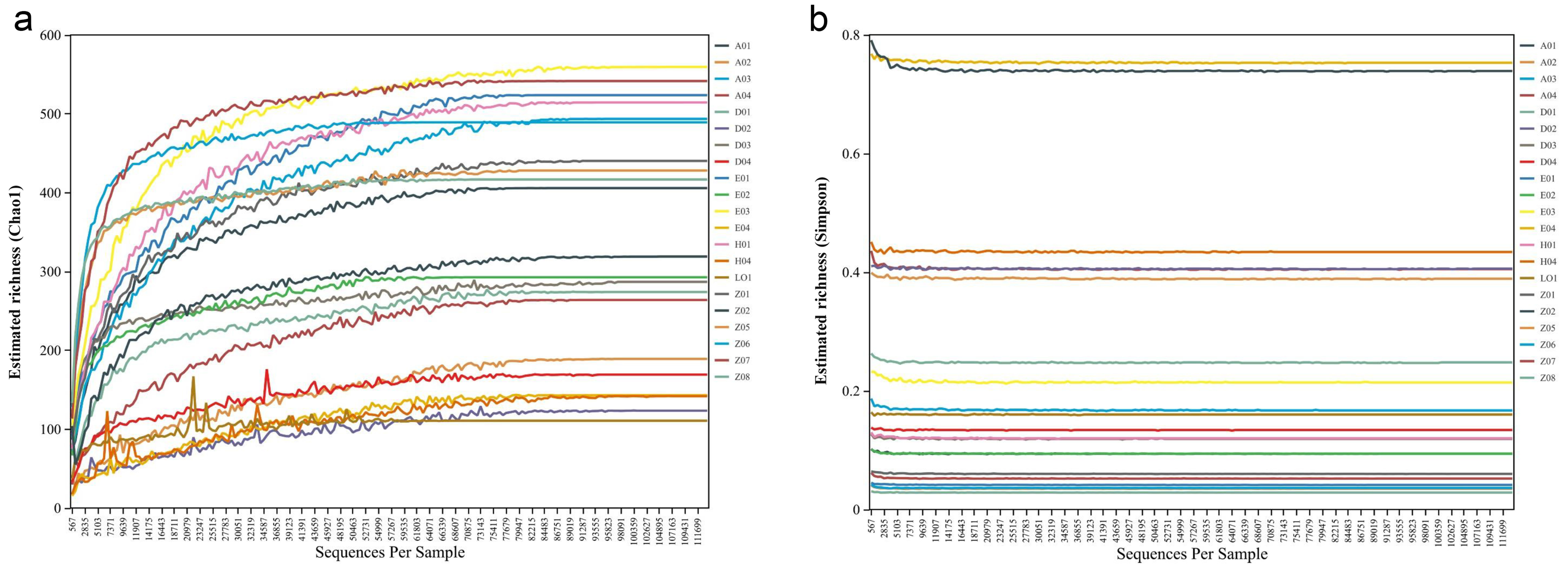

Supplement: Supplementary file 1 [file microorganisms-11-02359-s001.zip › Suppl. Figure S1.jpg]

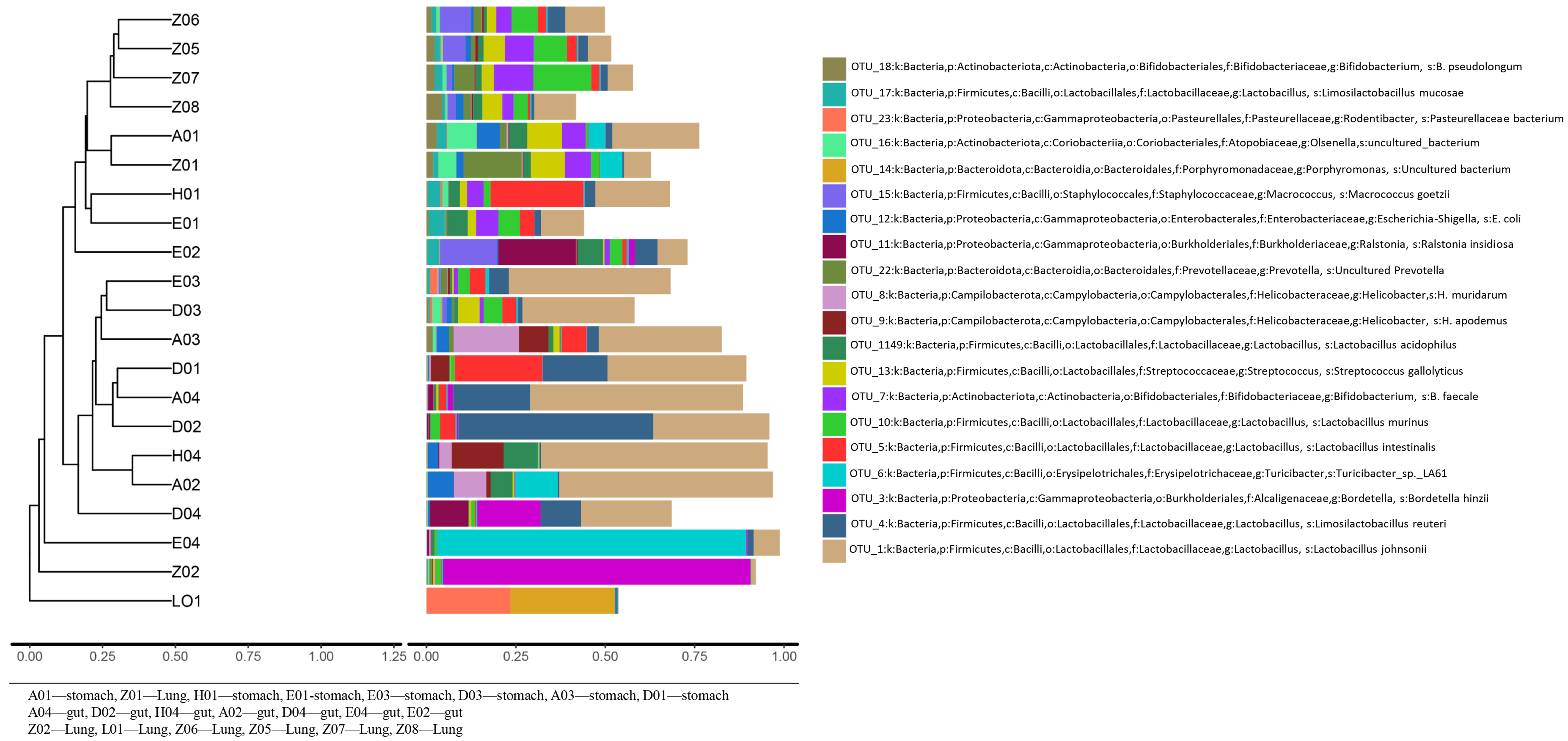

Supplement: Supplementary file 1 [file microorganisms-11-02359-s001.zip › Suppl. Figure S2 20 OTUs.jpg]

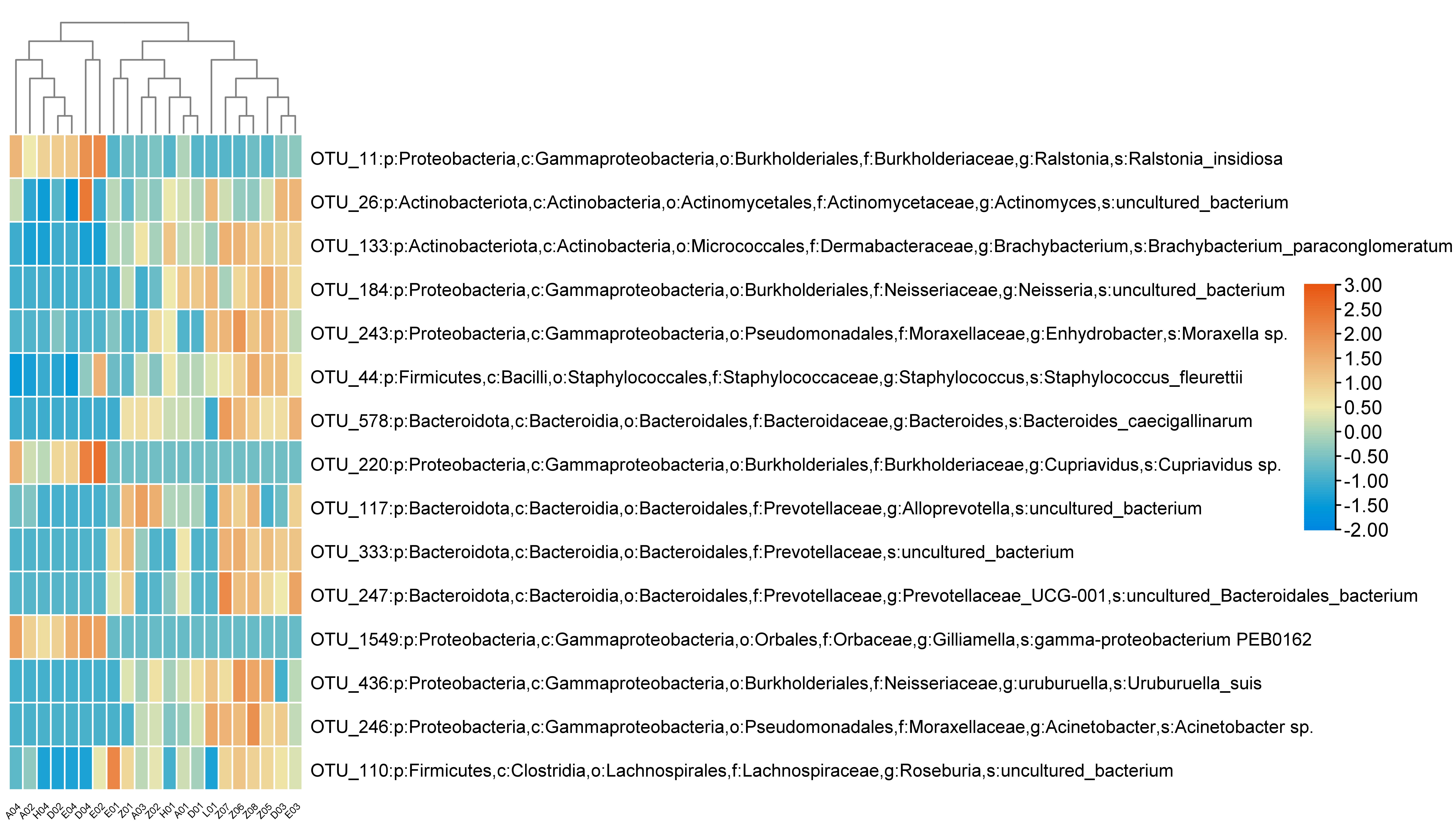

Supplement: Supplementary file 1 [file microorganisms-11-02359-s001.zip › Suppl. Figure S4. random forest analysis.jpg]
